# Application of Pigeon Myofibrillar Protein-Stabilized High Internal Phase Pickering Emulsions as a Sheep Tail Fat Substitute in Emulsion-Type Sausages

**DOI:** 10.3390/foods15213820

**Published:** 2026-10-26

**Authors:** Linghui Zhang, Xu Zhang, Yizhe Zhang, Wenhui Qi, Teng Li, Joel Rivadeneira, Yangliu Liu, Liwen Wang, Zhisheng Zhang

**Affiliations:** 1 College of Food Science and Technology, Hebei Agricultural University, Baoding 071000, China; zhanglinghui2008@126.com (L.Z.);; 2 School of Food Science and Biotechnology, Zhejiang Gongshang University, Hangzhou 310018, China; 3 Institute of Food Science and Technology, University of the Philippines Los Baños, Los Baños 4031, Philippines

**Keywords:** high internal phase Pickering emulsion, fat substitute, sheep tail fat, pigeon myofibrillar protein, emulsion-type sausage

## Abstract

To reduce the potential health risks associated with excessive intake of animal fat, emulsion-type sausages were prepared using a high internal phase Pickering emulsion (HIPPE) stabilized by pigeon myofibrillar protein (PMP) as a fat substitute, yielding fat-reformulated sausages. The effect of PMP concentration (0.5–2.5%, *w*/*v*) on the physical stability of HIPPEs was evaluated. Increasing PMP concentration improved the storage, centrifugal, and thermal stability of HIPPEs. Based on the stability results, HIPPE with 2.0% PMP was selected as a fat substitute to replace 50% or 100% of the sheep tail fat in emulsion-type sausages. Compared with the control, partial replacement (50%) of sheep tail fat with HIPPE maintained the cooking loss and emulsion stability of the sausages. However, when the sheep tail fat was completely replaced with HIPPE, these indicators improved significantly. Replacing sheep tail fat (either half or completely) with HIPPE significantly improved the rheological parameters of the meat batter. In addition, the water-holding capacity, lightness, and texture parameters (hardness and chewiness) of the sausages were significantly improved. In the sensory evaluation by a panel of food-science students, HIPPE addition positively affected the appearance, juiciness, texture, and overall acceptability of the sausages. Therefore, PMP-stabilized HIPPEs show potential for improving the technological quality of emulsion-type sausages and provide a basis for further exploration of vegetable oils in fat-reformulated meat products.

## 1. Introduction

Traditional emulsion-type sausages typically contain approximately 20–30% animal fat [1], which contributes to juiciness, energy, gel strength, and texture. Meanwhile, aromatics generated by lipolysis during food processing can enhance the unique flavor of the product, stimulating consumers’ appetites [2]. However, excessive intake of animal fat, particularly saturated fatty acids, has been associated with increased risks of obesity, cardiovascular disease, and other health problems [3,4]. With advocacy from the World Health Organization, products with reduced total fat content that maintain desired properties have gained increasing recognition. Nevertheless, directly reducing animal fat content usually compromises sensory properties (such as flavor intensity and tenderness) and the technological performance [5], making animal fat reduction a significant challenge. Consequently, researchers have widely investigated various strategies to reduce animal fat content in sausages [6].

The use of fat substitutes has emerged as a feasible strategy for reducing animal fat content while preserving desired quality. Researchers have explored proteins [7], polysaccharides [8], and protein-polysaccharide complexes [9] as fat substitutes. Another viable substitute is liquid vegetable oils, which are usually prepared into oleogels, emulsions/emulsion gels, microencapsulates, or lipid modifications before substitution [10,11]. These vegetable oil products can partially or fully replace animal fat in food while preserving sensory, flavor, and texture qualities [12]. Recently, high internal phase Pickering emulsions (HIPPEs) have emerged as a more promising alternative for replacing animal fat in meat products.

HIPPEs, typically with an internal dispersed phase volume fraction exceeding 74%, are highly concentrated emulsions stabilized by Pickering particles [13]. The Pickering particles form a rigid interfacial layer at the oil–water interface, enabling HIPPEs to resist droplet coalescence and other destabilizing factors. This unique structure endows HIPPEs with semi-solid, gel-like rheological characteristics and a high specific surface area similar to animal fats [14]. Animal adipose tissue consists mainly of adipocytes, whose cytoplasm contains lipid droplets. A small portion of cytoplasm remains at the cell edges, exhibiting flat, oval, or polygonal cell morphologies that mirror the oil droplet configuration typical of HIPPEs [15]. This structural resemblance suggests that HIPPEs are excellent candidates for fat replacement.

Proteins are widely used as Pickering stabilizers because of their amphiphilic nature and ability to adsorb at the oil–water interface. Among them, myofibrillar protein, accounting for 50–70% of total muscle protein, is an amphiphilic protein containing both hydrophilic and hydrophobic groups. It can adsorb at the oil–water interface, reduce surface tension, and disperse oil droplets uniformly within the aqueous phase, forming stable emulsions. These interfacial and emulsifying properties make myofibrillar protein a natural candidate for stabilizing HIPPEs. However, myofibrillar proteins from different animal sources vary significantly in structure and emulsifying performance [16,17], resulting in diverse HIPPE properties.

HIPPEs stabilized by plant proteins (e.g., pea protein, soy protein isolate) have been successfully applied as fat replacers in various food systems [18], including cookies [19], low-fat meatballs [20], and emulsion-type sausages [4,21]. For example, Yan et al. [19] used pea protein isolate-guar gum conjugate nanoparticles to stabilize HIPPEs and improve the sensory quality of emulsion-type sausages. Similarly, Wang et al. [4] incorporated soy protein isolate and sodium alginate-stabilized HIPPEs as fat substitutes in emulsion-type sausages, modulating aroma and taste attributes. These studies demonstrate the feasibility of HIPPEs as fat substitutes in sausages. However, animal-derived protein-stabilized HIPPEs remain much less investigated in sausage systems. In particular, pigeon myofibrillar protein (PMP) has not been investigated as a HIPPE stabilizer, and its interfacial behavior and effects on sausage quality are unknown. To the best of our knowledge, this is the first study to investigate PMP-stabilized HIPPEs and their application in emulsion-type sausages.

Here, the objective of this study was to investigate the stability of PMP-stabilized HIPPEs at different PMP concentrations and to evaluate their application as a partial or complete substitute for sheep tail fat in emulsion-type sausages. We hypothesized that increasing PMP concentration would improve the storage, centrifugal, and thermal stability of HIPPEs and that replacing sheep tail fat with PMP-stabilized HIPPEs would improve the technological quality of emulsion-type sausages while maintaining sensory acceptability. Therefore, this study investigated the physical stability of HIPPEs at different PMP concentrations. Based on the stability results, the optimal PMP concentration was selected and used as a fat substitute. Its impact on the quality of emulsion-type sausages when partially or fully replacing animal fat was then examined.

## 2. Materials and Methods

### 2.1. Materials and Samples

Fresh meat from the “Tianxiang No. 1” meat pigeon (*Columba livia*) (60–70 days old, 400–500 g body weight) was provided by a local slaughterhouse (Baoding, China). Fresh lamb hind leg meat and sheep tail fat were purchased from a local supermarket. The lamb meat was obtained from 6-month-old male Small-tailed Han sheep. Almond oil (pressed, 375 mL, product standard code: Q/SLCG 0002S) was purchased from Hebei Siluchenguang Oil Co., Ltd. (Handan, China). According to the manufacturer’s nutritional label, the oil contained 99.9% fat, with 68.9% monounsaturated fatty acids and 25.5% polyunsaturated fatty acids per 100 g. The density of almond oil was determined to be 0.91 g/mL at 25 °C. The almond oil was stored at 4 °C in the dark before use. Salt was purchased from Hebei Zhongyan Longxiang Salt Chemical Co., Ltd. (Xingtai, China). White pepper powder and ginger powder were purchased from Shandong Meiyitian Food Co., Ltd. (Dezhou, China). Potato starch was purchased from Daideitian Food Group Co., Ltd. (Wuhan, China). Salt, white pepper powder, ginger powder, potato starch, sodium tripolyphosphate, and ascorbic acid sodium were of food-grade quality. Nile Red and Nile Blue reagents were purchased from Shanghai Aladdin Biochemical Technology Co., Ltd. (Shanghai, China). Collagen protein intestine was purchased from Henan Wanbang Chemical Technology Co., Ltd. (Zhengzhou, China). Unless otherwise specified, deionized water and analytically pure chemical reagents were used in all experiments.

### 2.2. Experimental Design and Replication

All experiments were conducted in three independent batches unless otherwise stated. An independent batch refers to a complete replicate of the entire procedure, starting from a separate extraction of PMP from fresh pigeon meat, followed by independent preparation of HIPPEs, and ending with independently manufactured emulsion-type sausages for each treatment group. The three batches were prepared on different days using freshly obtained raw materials.

For each treatment group, three independent batches were produced, and within each batch, all four sausage treatment groups were prepared (one sausage per replicate measurement) and analyzed. Therefore, treatment observations generated from the same batch were not fully independent, and batch was treated as a blocking factor in the statistical analysis. For thermal stability, aliquots from the same HIPPE batch were exposed to the different temperatures (25, 60, 80, and 100 °C). Therefore, temperature was treated as a repeated-measures factor within each batch, and batch was included as a random factor. Except for sensory evaluation, where the individual panelist served as the experimental unit (*n* = 40), the experimental unit for all analyses was one independently prepared batch, with three independent batches (*n* = 3) per treatment. For each independent batch, measurements were performed in triplicate, and their mean was used as a single data point for statistical analysis. Technical replicates and subsamples were not treated as independent experimental units.

### 2.3. Preparation of PMP-Based HIPPEs and Emulsion-Type Sheep Sausages

#### 2.3.1. Preparation of PMP-Enriched Fraction

**Figure 1:**
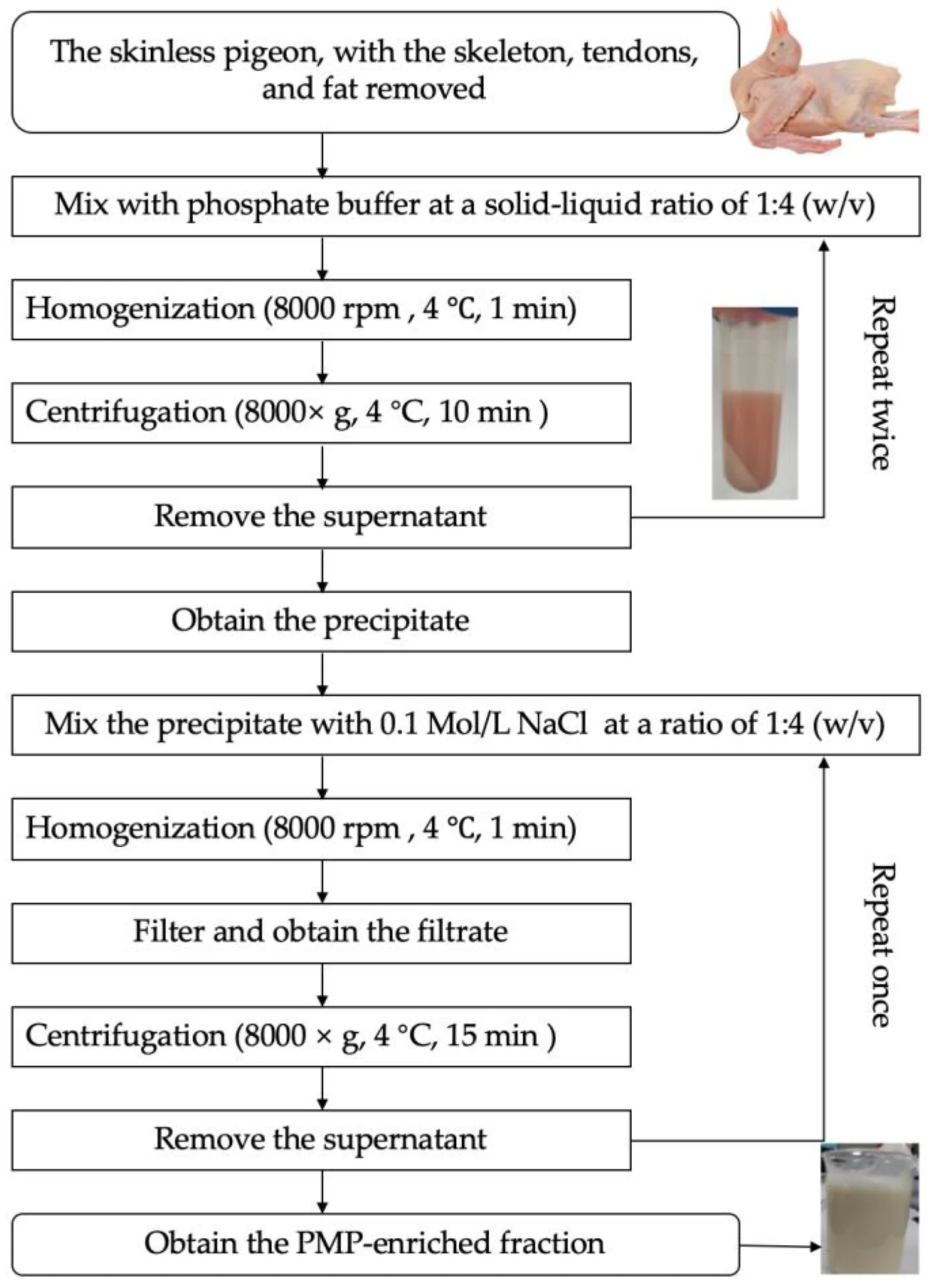
The schematic diagram of the processing steps of the PMP-enriched fraction.

The extraction of the PMP-enriched fraction followed the method described in a previous study [22] with some modifications. Figure 1 shows the schematic diagram of the extraction process. Fresh Pigeon without feathers was obtained. The skeleton, tendons, and fat were removed, and the raw meat was ground into a uniform meat paste using an MJ-LZ25Easy225 meat grinder (Guangdong Midea Household Appliance Manufacturing Co., Ltd., Foshan, China) at 4 °C. The meat paste was mixed with phosphate-buffered solution (0.1 mol/L NaCl, 2 mmol/L MgCl_2_, 10 mmol/L Na_2_HPO_4_/NaH_2_PO_4_, 1 mmol/L EDTA, pH = 7.0) at a solid–liquid ratio of 1:4 (*w*/*v*). The mixture was homogenized at 8000 rpm for 1 min using a homogenizer (D130, Wiggens GmbH, Straubenhardt, Baden-Württemberg, Germany) at 4 °C, then centrifuged at 8000× *g* for 10 min at 4 °C using a centrifuge (XZ-21K, Xiangzhi Centrifuge Instrument Co., Ltd., Changsha, China). The above homogenization and centrifugation steps were repeated twice, and the supernatant was removed. The resulting precipitate was mixed with 0.1 mol/L NaCl solution at a solid–liquid ratio of 1:4 (*w*/*v*), homogenized at 8000 rpm for 1 min at 4 °C, centrifuged at 8000× *g* for 15 min at 4 °C, and the supernatant was removed. The above homogenization and centrifugation steps were repeated once. The final precipitate was stored at 4 °C in a refrigerator (BCD-649WE, Qingdao Haier Co., Ltd., Qingdao, China) and used within 48 h after extraction. The final precipitate was not further purified and is referred to as a PMP-enriched fraction throughout this study. The protein content of the PMP-enriched fraction was determined by the bicinchoninic acid (BCA) assay (Section 2.4.1).

#### 2.3.2. Preparation of HIPPEs

The PMP-enriched fraction was dispersed in a buffer solution (0.6 mol/L NaCl, 10 mmol/L Na_2_HPO_4_/NaH_2_PO_4_, pH 7.0) and diluted to obtain PMP suspensions at different mass concentrations (0.5%, 1.0%, 1.5%, 2.0%, and 2.5%, *w*/*v*). The almond oil was mixed with the PMP suspension at an oil–water ratio of 3:1 (*v*/*v*), and then pre-emulsified for 30 s at 15,000 rpm using the D130 homogenizer. A crude emulsion was obtained and then ultrasonicated for 12 s using a high-intensity ultrasound processor (VCX750 Vibra-Cell, SONICS, Newtown, CT, USA) at 45% amplitude (power 337.5 W) in a pulse mode (on 3 s, off 18 s). During ultrasonication, the emulsion was placed in an ice-water bath to prevent overheating. Finally, PMP-stabilized HIPPEs were obtained.

#### 2.3.3. Preparation of Emulsion-Type Sheep Sausages

After removing the fat and tendons from the freshly slaughtered hind leg meat, it was placed in a meat grinder and minced at a low temperature (below 10 °C) for 1 min. Ascorbic acid sodium, sodium tripolyphosphate, and seasonings (salt, starch, white pepper powder, and ginger powder) were added to the minced meat mixture, along with half of the ice water. The mixture was minced again for 1 min. Then, the remaining ice water and sheep tail fat/almond oil/HIPPE were added to the meat mixture, and it was minced for 1 min. The sheep tail fat was trimmed of connective tissue, finely minced, and maintained at room temperature (20–25 °C) to achieve a paste-like consistency before incorporation into the meat batter. The meat mixture was injected into a 22 mm diameter collagen protein sausage casing using a manual sausage filler. A knot was manually tied with a cotton thread every 15 cm. Then, it was placed in a high-temperature steaming bag and heated in an 80 °C water bath for 20 min to ensure that the center temperature of the sausage reached 80 °C. Finally, the sausages were rapidly cooled to room temperature and vacuum-treated before being stored in the 4 °C refrigerator.

The total formulation mass was kept constant across all treatments (297.56 g per batch). Ice water was adjusted to compensate for the water introduced through the HIPPE. The total fat content was approximately equal across CON, S-AO, S-HH, and S-HW (60.00, 60.00, 59.28, and 58.56 g per batch, respectively), with a small difference (≤2.3%) arising from the density difference between almond oil and the aqueous phase.

The S-AO group was intentionally designed as a composition-matched, non-emulsified control for S-HW. Both groups had the same total formulation mass (297.56 g), similar total PMP content (~0.4 g), and similar total water content (~40 g). The key difference was that in S-AO, the almond oil and 2% PMP suspension were added directly to the meat batter, whereas in S-HW, the almond oil was pre-emulsified into a HIPPE before addition. Because the HIPPE was prepared at an oil-to-aqueous phase volume ratio of 3:1 (*v*/*v*), the actual oil content introduced through 80 g of HIPPE was approximately 58.6 g, slightly lower than the 60 g of almond oil added directly in S-AO. This small difference (approximately 2.3%) arose from the density difference between almond oil (0.91 g/mL) and the aqueous phase (~1.00 g/mL). This difference is unlikely to account for the large differences in technological quality observed between S-AO and S-HW, which are more likely related to the HIPPE structure itself. This design allowed us to distinguish the effects of ingredient composition from the effects of structuring the ingredients as a HIPPE.

The specific recipe for the emulsion-type sausages is shown in Table 1. The treatment groups were as follows. The CON group was the emulsion-type sausages with the sheep tail fat formula. The S-AO group was the emulsion-type sausages with almond oil directly replacing the sheep tail fat. The S-HH group was the emulsion-type sausages with HIPPE replacing half of the sheep tail fat (50% replacement). The S-HW group was the emulsion-type sausages with HIPPE replacing the whole amount of sheep tail fat (100% replacement).

### 2.4. Measurement of PMP

#### 2.4.1. Protein Content

The protein content of the PMP-enriched fraction was determined using the BCA assay kit (Solarbio, Beijing, China) according to the manufacturer’s instructions. Briefly, 25 μL of sample or standard was mixed with 200 μL of working reagent in a 96-well plate and incubated at 37 °C for 30 min. The absorbance was measured at 562 nm using a microplate reader (SpectraMax i3x, Molecular Devices, San Jose, CA, USA). Bovine serum albumin (BSA) was used as the standard, and a standard curve was constructed over 0–2000 μg/mL. The protein purity was calculated as the ratio of protein content to the total dry weight of the PMP-enriched fraction. The protein content was expressed as % (*w*/*w*) on a dry basis. The analysis was performed in triplicate.

#### 2.4.2. AFM Analysis of PMP

The morphology of PMP was characterized using an atomic force microscope (AFM, Dimension Icon Corporation, Bruker, Billerica, MA, USA) in tapping mode. A dilute PMP suspension (0.01%, *w*/*v*) was deposited onto a freshly cleaved mica surface and air-dried at room temperature. The AFM images were collected at a scan size of 1.0 μm × 1.0 μm with a scan rate of 1 Hz. The AFM images were flattened and analyzed using NanoScope Analysis software (version 1.8, Bruker Corp., Santa Barbara, CA, USA). Three independent PMP batches were analyzed, and one representative image is shown.

#### 2.4.3. SDS-PAGE Analysis

SDS-PAGE was performed as described previously, with slight modifications. The original PMP, interfacial-adsorbed PMP (extracted from the HIPPE interfacial layer), or unadsorbed PMP (remaining in the aqueous phase) was adjusted to a concentration of 1 mg/mL. The adjusted suspensions were mixed with loading buffer at a 4:1 (*v*/*v*) ratio and heated in boiling water for 5 min. For SDS-PAGE analysis, 4 μL of the marker was added to the first lane, and 15 μL of the mixture was added to the remaining lanes, which were run at 80 V and then at 110 V until the tracking dye reached the bottom. The samples were stained with Coomassie Brilliant Blue R250 for 4 h and decolorized with deionized water until the bands were clear.

### 2.5. Characterization of HIPPEs

#### 2.5.1. Analysis of Emulsion Type

Following the method described in previous studies, the prepared HIPPE was dropped into the almond oil phase or water phase, and droplet dispersion was observed to infer the type of HIPPE. If the emulsion droplets rapidly dispersed in the water phase and did not aggregate in the oil phase, the prepared emulsion was classified as an oil-in-water Pickering emulsion. Conversely, if the droplets did not disperse in the water phase and aggregated in the oil phase, it was classified as a water-in-oil Pickering emulsion.

#### 2.5.2. Measurement of Average Droplet Size

The microstructure of the HIPPEs was observed using an optical microscope (ECLIPSE Ci, Nikon, Tokyo, Japan). The emulsions were diluted 100-fold with deionized water and then observed at 400× magnification. Three independently prepared HIPPE batches were used for droplet-size analysis (*n* = 3 independent batches). For each batch, three aliquots were prepared, and one microscopy image was captured per aliquot, resulting in three images per batch. The droplet size of the emulsion was analyzed using ImageJ software (version 1.53, National Institutes of Health, Bethesda, MD, USA). For each image, three non-overlapping regions were randomly selected using the “Random Selection” function or by dividing the image into a grid and randomly choosing squares. Within each region, all clearly distinguishable droplets were measured using the line-measurement tool, with the scale calibrated against a stage micrometer. Droplets that were overlapping, aggregated, or partially outside the region were excluded from measurement. For each image, at least 200 droplets were measured. For each independent batch, the mean droplet size was calculated from all measured droplets and used as a single data point for statistical analysis. Therefore, the experimental unit was the independent HIPPE batch (*n* = 3). The individual droplets and image fields were treated as technical replicates, not as independent experimental units.

#### 2.5.3. Microstructure Measurement

The confocal laser scanning microscope (CLSM) images of the samples were acquired using an LSM900 CLSM (Carl Zeiss AG, Oberkochen, Baden-Württemberg, Germany), following a previous study [23] with minor modifications. The Pickering emulsion was diluted 100-fold with deionized water. The dilution (5.0 mL) was stained with 100 μL of Nile Blue (0.1%, *w*/*v*, in anhydrous ethanol) and 100 μL of Nile Red (0.1%, *w*/*v*, in anhydrous ethanol). The measurement parameters are set as follows. The excitation wavelengths were set to 488 nm (the Nile Red channel) and 633 nm (the Nile Blue channel). The detector gains were set to 600–700 (the Nile Red channel) and 700–800 (the Nile Blue channel). The samples were observed at 400× magnification.

#### 2.5.4. HIPPE Physical Stability

In this study, HIPPEs were considered stable when no visible oil-phase separation occurred, and the change in average droplet size was ≤10% compared with the fresh sample. These criteria were applied consistently across all stability experiments.

Freshly prepared HIPPEs (about 10 mL) were stored at 4 °C for 30 days in sealed glass vials. The macroscopic appearance, microstructure, and average droplet size were recorded at 0 and 30 days.

The freshly prepared HIPPE was centrifuged at 8000× *g* for 10 min. Then, its macroscopic morphology was observed to check for stratification, oil separation, and emulsion coagulation after centrifugation, thereby evaluating the centrifugal stability of the high-inclusion Pickering emulsion. For HIPPEs with different PMP concentrations, the creaming index and oil yield after centrifugation were calculated, serving as quantitative indicators for evaluating the centrifugal stability of HIPPEs. The emulsion was centrifuged at 4000× *g* for 5 min at 25 °C; the oil and water were separated from the emulsion, and the ratios of the weight of the separated oil and water to the initial weight of the HIPPE were calculated. Oil yield was the ratio of the weight of the oil centrifuged out to the initial weight of the HIPPE. The creaming index and oil yield of HIPPE were calculated according to the following Equations (1) and (2), respectively:

(1) $$\mathrm{Creaming index}\;\left(\%\right)=\frac{W_{1}-W_{3}}{W_{1}-W_{0}}\times 100$$

(2) $$\mathrm{Oil yield}\;\left(\%\right)=\frac{W_{1}-W_{2}}{W_{1}-W_{0}}\times 100$$

where *W*_0_ represents the mass of the empty centrifuge tube (g), *W*_1_ represents the mass of the centrifuge tube with the sample (g), *W*_2_ represents the mass of the centrifuge tube with the sample after removing the liquid oil, and *W*_3_ represents the mass of the centrifuge tube with the sample after removing the oil and the bottom water.

Freshly prepared HIPPE (10 mL) was sealed in a 20 mL sample bottle. The fresh HIPPE was placed in water baths at 30 °C, 60 °C, 80 °C, and 100 °C for 30 min of continuous heating, respectively. The samples were then rapidly cooled to room temperature. The macroscopic morphology and microstructure of the samples were observed and analyzed.

### 2.6. Measurement of the Sausages

#### 2.6.1. Rheological Properties of Meat Batter

The rheological parameters of meat batter were determined using a Discovery HR 20 rheometer (TA Instruments, New Castle, DE, USA) equipped with a 40 mm parallel plate with a 1.0 mm gap at 25 °C. After 5 min of equilibration, the apparent viscosities of the samples were recorded over a shear rate range of 0.01 to 100 s^−1^. After determining the linear viscoelastic range, the storage modulus (G′) and loss modulus (G′′) of the samples were recorded at a strain of 0.1% (within the linear viscoelastic range) over a frequency range of 0.1 to 10 Hz.

#### 2.6.2. Proximate Analysis

Water and ash contents of the emulsion-type sausages were estimated according to the Association of Official Analytical Chemists methods (AOAC 2000). Protein was measured by the Kjeldahl method. Total fat content was determined in duplicate by Soxhlet extraction with diethyl ether as the solvent.

#### 2.6.3. pH Measurement

The cooked sausage (about 5 g) was crushed and mixed with deionized water at a 1:10 (*w*/*v*) ratio. The mixture was homogenized using the homogenizer at 8000 rpm for 1 min. The sample was then filtered, and the filtrate pH was measured using a pH meter (Starter 3100, Ohaus Instrument Co. Ltd., Shanghai, China). The analysis of each sample was performed in triplicate.

#### 2.6.4. Measurement of Cooking Loss

After accurately weighing and recording the samples from the different treatment groups prepared in step 2.3 (*W*_0_), the sausage samples were placed in high-temperature sterilization bags and heated in an 80 °C-water bath for 20 min. The sausage samples were then cooled at room temperature and weighed again (*W*_1_). The formula for calculating the cooking loss was as follows:

(3) $$\mathrm{Cooking loss}\;\left(\%\right)=\frac{W_{0}-W_{1}}{W_{0}}\times 100$$

#### 2.6.5. Measurement of Water-Holding Capacity (WHC)

The WHC of sausages was determined according to the method reported in our previous study [24]. Approximately 10 g of each sample was accurately weighed (*W*_1_) and placed in a conical flask. The conical flask was closed and then heated in a water bath at 90 °C for 10 min. After cooling to room temperature, it was placed in a 50 mL centrifuge tube, with cotton wool at the bottom of each tube. The sample was then centrifuged at 4 °C at 9000 rpm for 10 min. The sausage sample was reweighed (*W*_2_). The formula for calculating WHC is as follows:

(4) $$\mathrm{WHC}\;\left(\%\right)=(1-\frac{W_{1}-W_{2}}{W_{\mathrm{water}}})\times 100\;$$

where *W*_1_ is the initial sample weight before centrifugation (g), *W*_2_ is the sample weight after centrifugation (g), and *W_water_* is the total water content of the sample (g), calculated as the product of the sample weight and its moisture content (Section 2.6.2).

#### 2.6.6. Emulsion Stability

Emulsion stability was determined using a previously reported method [25], with some modifications. Approximately 20 g of raw sausage mince was accurately weighed and recorded as *W*_1_. The sample was placed in a 50 mL centrifuge tube and heated in a water bath at 80 °C for 20 min, then centrifuged at 10,000× *g* for 5 min. After centrifugation, the supernatant was carefully removed, and the weight of the remaining precipitate was recorded as *W*_2_. The supernatant was transferred to a crucible and dried at (100 ± 2) °C until constant weight. The dried supernatant was then subjected to Soxhlet extraction to determine the fat content, which was recorded as *W*_3_. The emulsion stability of the sausage was generally expressed by total expressible fluid (TEF) and fat in TEF. The calculation formulas were as follows (Equations (5) and (6)).

(5) $$\mathrm{TEF}\;\left(\%\right)=\frac{W_{1}-W_{2}}{W_{1}}\times 100$$

(6) $$\mathrm{fat in TEF}\;\left(\%\right)=\frac{W_{3}}{W_{1}-W_{2}}\times 100$$

where *W*_1_ is the weight of raw sausage mince (g), *W*_2_ is the weight of the precipitate remaining after removal of the supernatant (g), and *W*_3_ is the weight of the fat extracted from the dried supernatant by Soxhlet extraction (g).

#### 2.6.7. Color Parameters of Cooked Sausages

The color parameters of cooked sausages were measured according to the CIELAB color space (CIE 1976) using a CR-400 hand-held colorimeter (Konica Minolta Investment Ltd., Osaka, Osaka Prefecture, Japan) with a D65 illuminant and a 2° standard observer. The aperture size was 8 mm. The colorimeter was calibrated before measurement, and the L*, a*, and b* values of the standard white color chart were 93.15, 0.12, and 3.09, respectively. Here, L* represented lightness, ranging from 0 (black) to 100 (white). A higher L* value indicates a lighter color. a* represented redness, ranging from negative (green) to positive (red). A higher a* value indicates a redder color. b* represented yellowness, ranging from negative (blue) to positive (yellow). A higher b* value indicates a more yellow color. The cooked emulsion-type sausages were cut into uniform cylinders 10 mm in thickness. Measurements were taken directly on the freshly cut surface of each sample, with no glass covers, films, or other optical accessories placed between the instrument and the sample. The L*, a*, and b* values at the center of each sample were measured. All measurements were performed against a white background to standardize background interference across all formulations. The total color difference (ΔE*) for each sample relative to the control sausage was calculated using Equation (7) [26]:

(7) $$\Delta E*=\sqrt{{\left(∆L*\right)}^{2}+(∆{a*)}^{2}+(∆{b*)}^{2}}$$

where ΔL* = L_sample_* − L_CON_*, Δa* = a_sample_* − a_CON_*, and Δb* = b_sample_* − b_CON_*. L_CON_*, a_CON_*, and b_CON_* are the L*, a*, and b* values of the control sausages (CON), respectively. L_sample_*, a_sample_*, and b_sample_* are the L*, a*, and b* values of the sausages measured, respectively.

#### 2.6.8. Texture Profile Analysis (TPA)

The casing of the cooked sausage was removed. The sausages from different treatment groups were cut into cylinders 10.0 mm high and 22.0 mm in diameter, ensuring that each sample had the same dimensions and a smooth surface. The cylindrical samples were equilibrated at room temperature (25 °C) for 30 min before measurement. TPA was performed using a TMS-Touch texture analyzer (FTC company, Sterling, VA, USA) equipped with a 36 mm aluminum cylindrical probe (P/36R). A two-cycle compression test was performed along the axial direction. The pre-test, test, and post-test rates were all 2.0 mm/s, with a 5.0 s interval between the two compression cycles. The compression ratio was set to 60% of the original sample height. The trigger force was 0.05 N. The data acquisition rate was 200 points/s. The following parameters were calculated from the force–time curves: hardness (N), cohesiveness (ratio), springiness (mm), and chewiness (N·mm). Three independently manufactured sausage batches were used for TPA (*n* = 3 independent batches). For each batch, three cylindrical samples were measured, and their mean was used as a single data point for statistical analysis.

#### 2.6.9. Sensory Evaluation

The sensory evaluation of emulsion-type sheep sausages with different formulas was conducted [21]. Forty panelists (20 males and 20 females) were recruited from food science students at Hebei Agricultural University (*n* = 40). The inclusion criteria were: (1) aged 18–30 years; (2) no known allergies to meat, almond, or related products; (3) no reported taste or smell disorders; (4) willing to provide written informed consent. The exclusion criteria were: (1) smoking; (2) pregnancy; (3) any medical condition affecting sensory perception. All panelists were briefed on the study purpose and evaluation procedure before the test, and written informed consent was obtained.

Cooked sausages were cut into circular slices of uniform thickness (2 cm) at room temperature. Each sample was placed in a disposable, odorless plastic container labeled with a random three-digit code. The coding was different for each panelist to prevent carry-over effects from previous evaluations. Samples were served at 22 ± 2 °C. Each panelist evaluated all four formulations (CON, S-AO, S-HH, and S-HW) in a single session. The order of sample presentation was randomized and balanced across panelists using a Latin square design. Hence, each formulation appeared in each position approximately equally often to minimize order and carry-over effects. Panelists were blinded to the formulation identity and the purpose of the study. Samples were coded and presented under uniform lighting conditions. Panelists were not informed which sample contained sheep tail fat, almond oil, or HIPPE. Evaluations were performed in individual booths under white fluorescent lighting at 22 ± 2 °C. Panelists rinsed their mouths with water and ate unsalted crackers between samples to cleanse the palate and remove residual effects.

Panelists rated each sample for flavor, juiciness, appearance, texture, and overall acceptability using a nine-point hedonic scale (1 = extreme dislike, 9 = absolutely like). No specific training was provided, as the panelists were familiar with sensory evaluation methods through their coursework. However, the evaluation procedure and scale meaning were explained to all panelists before the test. Sensory data are expressed as the mean ± standard deviation (SD) of 40 panelists (*n* = 40). The experimental unit was the individual panelist.

### 2.7. Statistical Analyses

All data are shown as mean ± SD of three independent batches (*n* = 3), unless otherwise specified. For each independent batch, measurements were performed in triplicate and averaged before statistical analysis. Technical replicates and subsamples were not treated as independent experimental units. Statistical analyses were performed using SPSS 30.0 (IBM Corp., Armonk, NY, USA), and a significance level of *p* < 0.05 was used for all statistical tests. The statistical analysis used independent experimental units to avoid pseudoreplication. Graphical representations were drawn using Origin 2026 (OriginLab, Northampton, MA, USA).

Before parametric analysis, the normality of residuals was assessed using the Shapiro–Wilk test, and the homogeneity of variances was assessed using Levene’s test. When the assumptions were met (*p* > 0.05), parametric analysis of variance (ANOVA) was used. When the assumptions were violated, data were either log-transformed or analyzed using the Kruskal–Wallis test followed by Dunn’s post hoc test with Bonferroni correction.

Batch was first tested as a random factor in a linear mixed-effects model. The batch effect was not statistically significant (*p* > 0.05) for the analyzed indicators, and the variance component associated with batch was negligible. Therefore, one-way ANOVA followed by Duncan’s multiple range test was used for the final analysis of the single-factor experiments (proximate composition, pH, cooking loss, WHC, emulsion stability, color, texture profile, and centrifugal stability of HIPPEs).

For the thermal stability of HIPPEs, batch was also tested as a random factor. Since the batch effect was not significant (*p* > 0.05), a two-way ANOVA was used with PMP concentration and temperature as fixed factors, including their interaction (PMP concentration × temperature). When a significant interaction was observed, simple effects analysis was performed; otherwise, main effects were interpreted. Multiple comparisons were conducted using Duncan’s multiple range test.

Sensory data were analyzed using a mixed-effects model with formulation as a fixed factor and panelist as a random factor, to account for the dependence among repeated evaluations by the same panelist. The mixed model was selected because each panelist evaluated all four formulations, creating a repeated-measures structure. Because the residuals met the assumptions of normality and homoscedasticity (Shapiro–Wilk test, *p* > 0.05; Levene’s test, *p* > 0.05), a nonparametric approach was not used. Multiple comparisons among formulations were performed using Bonferroni-corrected pairwise comparisons. The individual panelist served as the experimental unit (*n* = 40).

## 3. Results

### 3.1. Characterization of PMP

#### 3.1.1. Composition of the PMP Preparation

**Figure 2:**
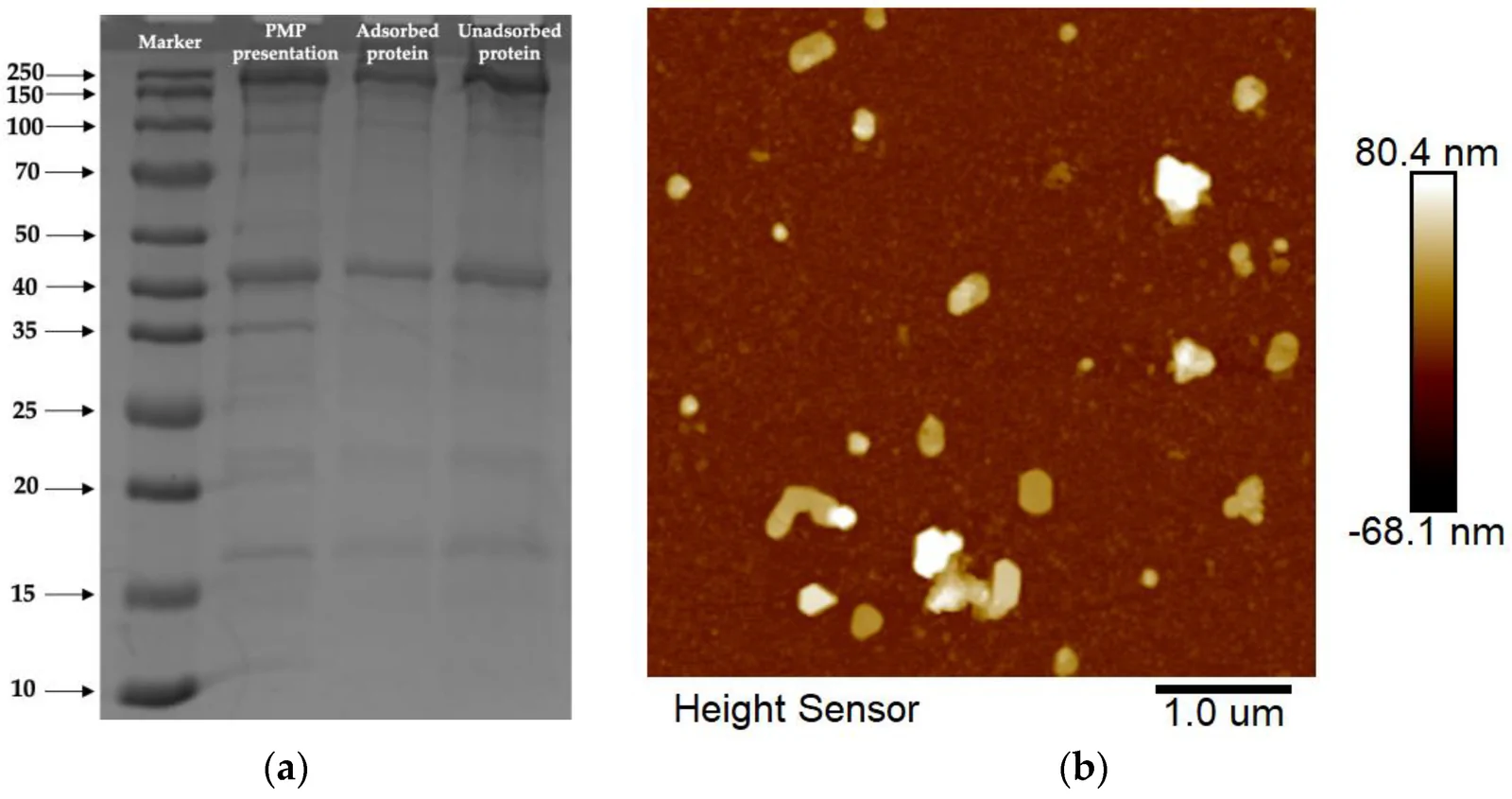
SDS−PAGE profile of the PMP−enriched fraction, interfacial-adsorbed protein, and unadsorbed protein (**a**), and AFM image of the PMP−enriched fraction (**b**). One representative image from three independent batches is shown.

The SDS-PAGE profile of the PMP preparation is shown in Figure 2a. The gel showed characteristic bands corresponding to myosin heavy chain (~200 kDa), paramyosin (~100 kDa), actin (~43 kDa), and tropomyosin (~35 kDa), confirming that the PMP preparation was enriched in myofibrillar proteins. No dominant band corresponding to sarcoplasmic proteins (e.g., myoglobin, ~17 kDa) was observed, indicating that the washing steps effectively removed most sarcoplasmic proteins and the PMP-enriched fraction was enriched in myofibrillar proteins. The protein content of the PMP-enriched fraction was (81.01 ± 0.92) % (*w*/*w*, dry basis), as determined by the BCA assay. This value is within the typical range reported for myofibrillar protein preparations (75–90%) [27]. The remaining components (approximately 19%) likely include ash and residual lipids. The interfacial-adsorbed PMP and unadsorbed PMP showed a band pattern similar to that of the bulk PMP, indicating that PMP adsorbed at the oil–water interface. These results support PMP particle adsorption at the oil/water interface.

The PMP-enriched fraction was used immediately after extraction. However, the stability (e.g., storage stability, thermal stability) of the PMP-enriched fraction itself was not separately investigated, and this should be investigated in future work.

#### 3.1.2. AFM Image of PMP

The morphology of PMP was characterized by AFM (Figure 2b). PMP formed discrete spherical or oval particles. This is consistent with the particulate nature of myofibrillar proteins, which can self-assemble into nanoscale particles under appropriate conditions. The heights of the PMPs ranged from approximately 20 to 80 nm, and the diameters ranged from approximately 20 to 50 nm. These dimensions are consistent with the typical size of myofibrillar protein aggregates reported in the literature. The particles were well distributed on the mica surface, with only minor aggregation in some regions. This suggests that the PMP suspension was well dispersed before deposition. This result indicated that PMP can exist in particulate form at the nanoscale, which is a prerequisite for Pickering stabilization. However, AFM analysis was performed on dried PMP samples, not on the oil–water interface. Therefore, AFM alone cannot confirm that PMP adsorbs at the interface in a particulate form.

### 3.2. Characterization of the HIPPEs

#### 3.2.1. Macroscopic Feature and Microstructure of HIPPEs

**Figure 3:**
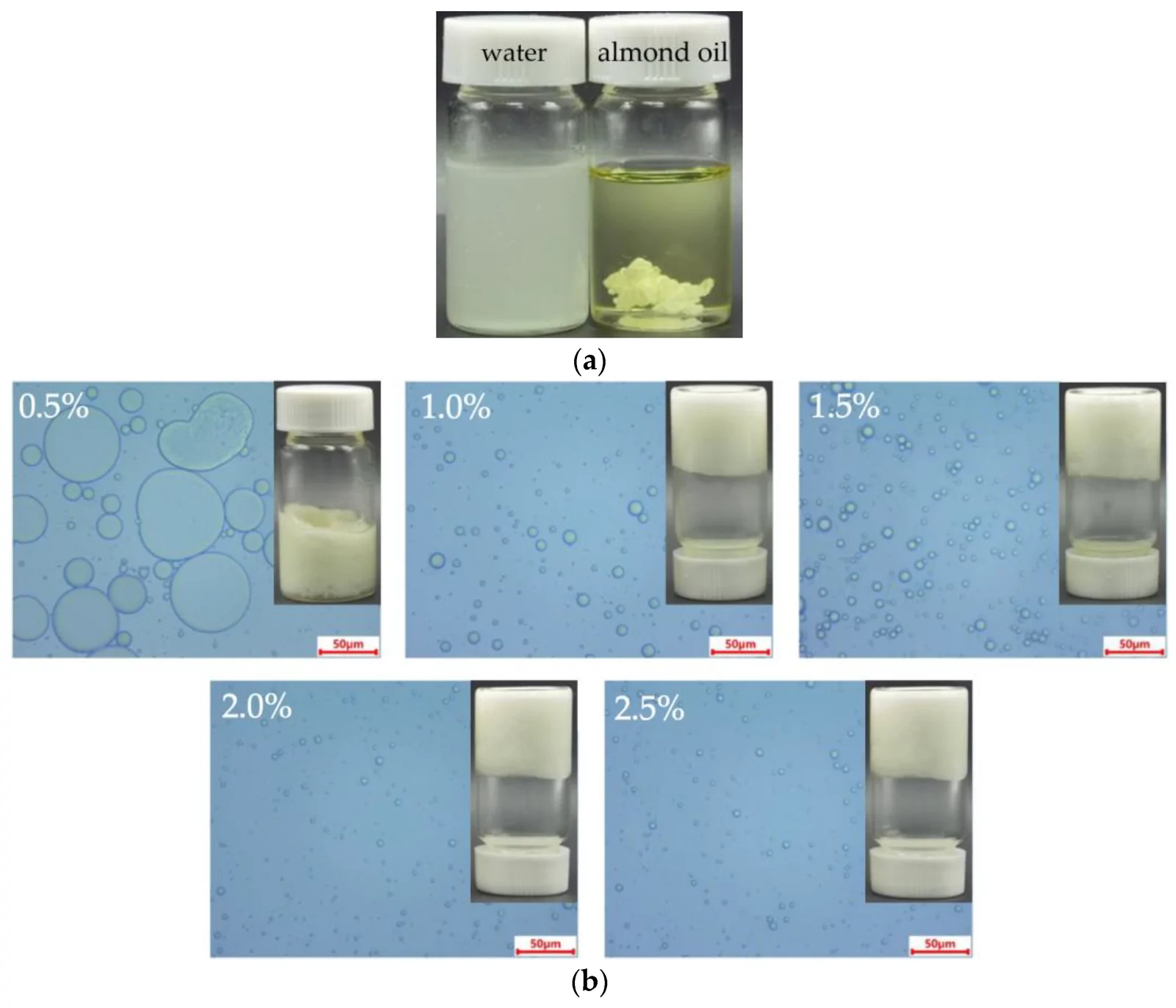
The dispersion state of PMP-based HIPPE droplets after being dropped into the water (left)/almond oil (right) system (**a**). Optical microscope image and macroscopic features of PMP-stabilized HIPPEs with different PMP concentrations (0.5%, 1.0%, 1.5%, 2.0%, and 2.5% *w*/*v*) (**b**). One representative image from three independent batches is shown.

As shown in Figure 3a, when dropped into deionized water, the PMP-based HIPPE dispersed rapidly. In contrast, when dropped into almond oil, the emulsion did not disperse and remained in its original shape. This phenomenon clearly indicated that the HIPPE stabilized by PMP belongs to an oil-in-water (O/W) type emulsion. This result is consistent with the previous report [28] that protein-based emulsions are all oil-in-water emulsions.

Figure 3b shows the macroscopic morphology of the stable HIPPEs with different concentrations of freshly prepared PMP (0.5%, 1.0%, 1.5%, 2.0%, and 2.5%). The HIPPE with 0.5% PMP showed obvious phase separation. An unencapsulated oil phase formed in the upper layer of the emulsion, and a water phase layer precipitated at the bottom, indicating that 0.5% PMP may be insufficient to prepare stable HIPPEs. This result may be due to the relatively low concentration of solid particles (PMP) in the system. The particles were insufficient to encapsulate at the oil–water interface and maintain emulsion physical stability, which consequently led to phase separation. For HIPPEs with PMP concentrations ranging from 1.0% to 2.5%, all showed uniform dispersion with a milky white color, and no obvious phase separation occurred. When the emulsion was inverted, the HIPPEs remained in their original state without flowing, preliminarily indicating that the emulsions were relatively stable.

The droplet sizes of the HIPPE prepared with a low PMP concentration (0.5%) were larger and relatively uneven (Figure 3). This phenomenon may be due to insufficient PMP adsorption at the oil–water interface, resulting in a weak interfacial film. When oil droplets collided, the emulsion droplets aggregated and formed larger droplets. When the PMP concentration is 0.5%, it is difficult to form a stable emulsion. Therefore, subsequent research mainly focused on HIPPE characteristics at PMP concentrations of 1.0% or higher. As the concentration of PMP increased (1.0–2.5%), the droplet sizes of the emulsion gradually decreased, and the droplet distribution became increasingly uniform. These results may be mainly attributed to the fact that, as the particle concentration increased, sufficient PMP was adsorbed at the oil–water interface. A large amount of PMP adsorption at the oil–water interface generated steric hindrance. This resistance could effectively reduce interfacial tension, thereby preventing droplets from aggregating [29] and ultimately enabling the system to form smaller emulsion droplets.

**Figure 4:**
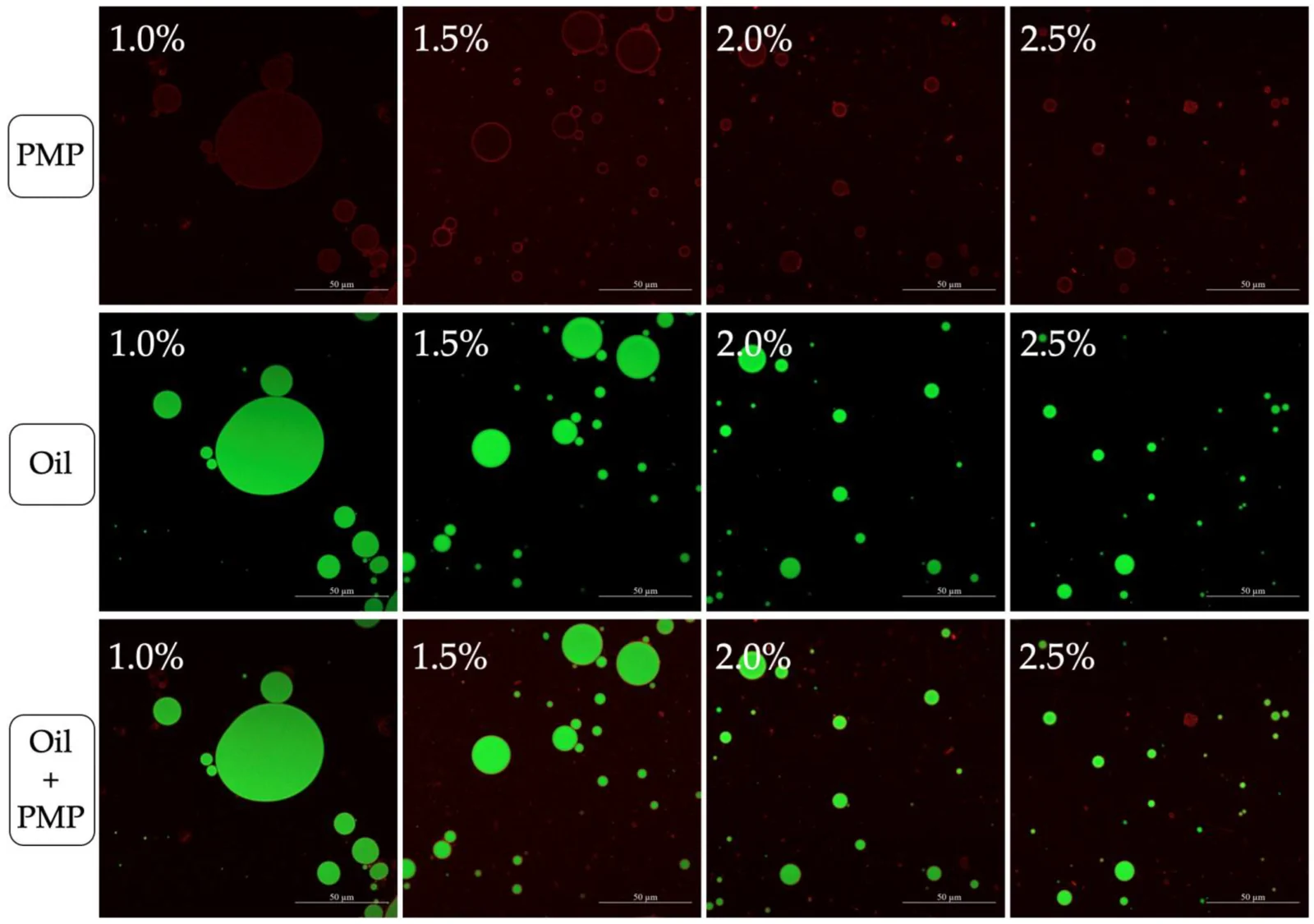
CLSM images of PMP-stabilized HIPPEs with different PMP concentrations (1.0%, 1.5%, 2.0%, and 2.5% *w*/*v*). One representative image from three independent batches is shown. The scale bar is 50 μm.

The microstructure of the HIPPE interface was further evaluated using CLSM. The green oil phase appeared as spherical droplets, and the red PMPs were distributed around the periphery of the oil droplets, forming a PMP-rich interfacial layer (Figure 4). This phenomenon was also consistent with an O/W type emulsion. As PMP concentration increased, droplet size decreased and became more uniform. The interfacial layer appeared thicker, suggesting that higher PMP concentration improved interfacial structure and contributed to emulsion physical stability.

AFM analysis confirmed that PMP can form nanoscale particles, and SDS-PAGE and CLSM analyses confirmed the adsorption of PMP at the oil–water interface. These results are consistent with Pickering-type stabilization, in which PMPs adsorb at the oil–water interface and form a dense interfacial layer. However, these techniques do not allow us to definitively distinguish between Pickering-type stabilization and conventional protein-stabilized emulsification. The formation of a gel-like interfacial layer was inferred from CLSM images but not directly measured.

**Figure 5:**
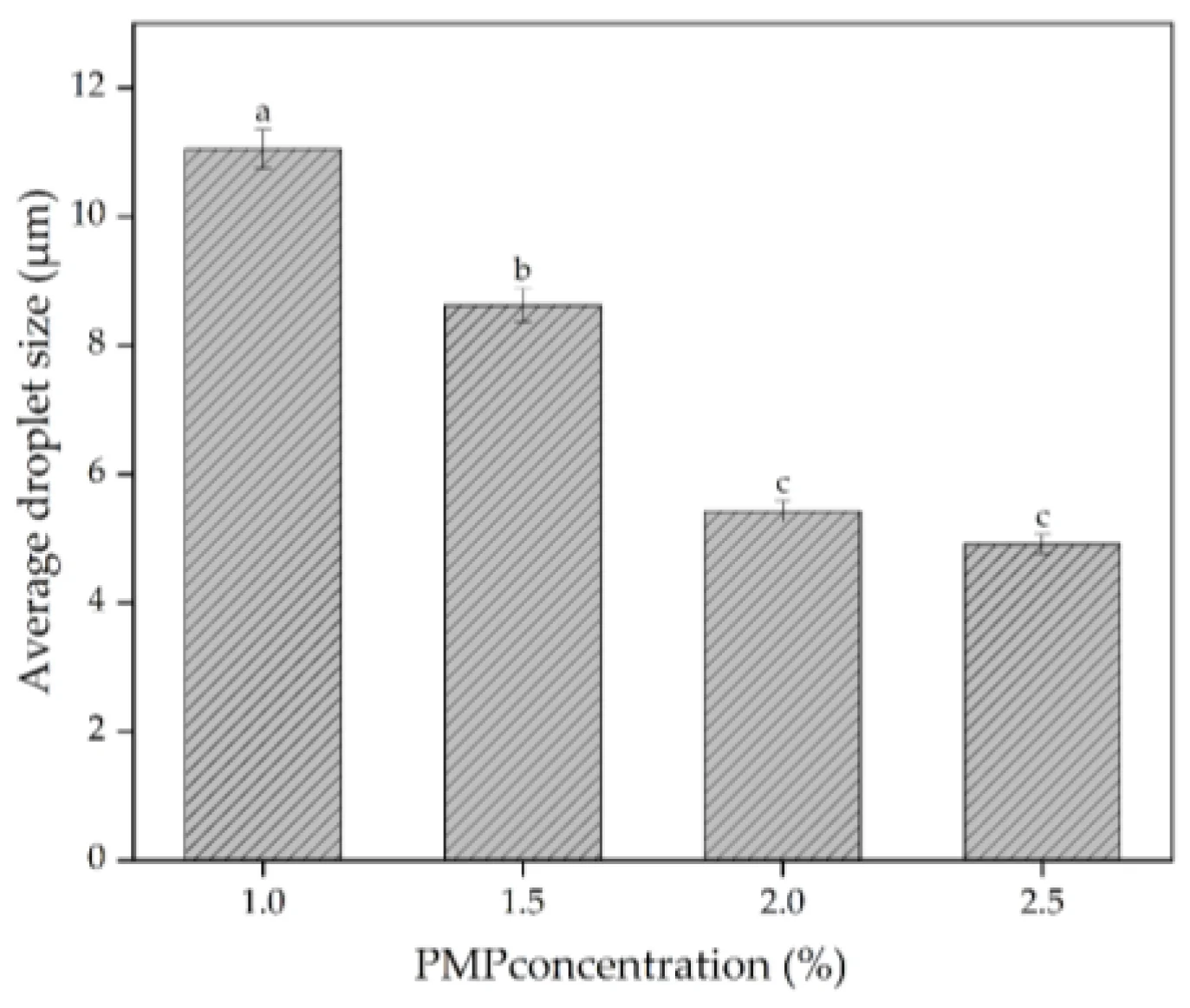
The average droplet size of PMP-stabilized HIPPEs at different PMP concentrations (1.0%, 1.5%, 2.0%, and 2.5% *w*/*v*). Data are expressed as the mean ± SD of three independent HIPPE batches (*n* = 3). Different lowercase letters indicate significant differences among HIPPEs under different PMP concentrations (*p* < 0.05).

The average droplet size of HIPPEs was measured using three independently prepared batches (*n* = 3). For each batch, at least 200 droplets were measured, and the mean was used as a single data point. As shown in Figure 5, the average droplet size of HIPPEs with a PMP concentration of 1.0% was the largest (11.05 μm). As PMP concentration increased from 1.5% to 2.0%, the average droplet size of HIPPE decreased significantly (*p* < 0.05). However, when the concentration increased further, droplet size did not change significantly. This result may have occurred because, when ultrasound broke the oil phase into small droplets, newly exposed interfaces required rapid PMP adsorption to reduce interfacial tension and form a physical barrier. At low PMP concentrations, the number of PMPs was insufficient to cover the newly formed oil–water interface fully. As PMP concentration increased, more particles adsorbed onto the interface per unit time, reducing interfacial tension more rapidly and decreasing the likelihood of droplet coalescence after homogenization. Therefore, as PMP concentration increased from 0.5% to 2.0%, the interfacial film became increasingly dense, effectively preventing droplet merging and significantly reducing the average droplet size of HIPPEs. At PMP concentrations of 2.0% and 2.5%, the average droplet sizes of HIPPE were 5.42 μm and 4.91 μm, respectively, with no statistically significant difference (*p* > 0.05). At a PMP concentration of 2.0%, sufficient PMPs adsorbed onto the oil–water interface to form a dense adsorption layer that nearly completely covered the interface. The densely packed PMPs on the interface created steric hindrance, effectively preventing droplet collisions and subsequent coalescence, thus reducing HIPPE droplet size. Beyond this point, additional PMPs could not adsorb onto the interface and remained free in the aqueous phase. Because of steric hindrance and interfacial rigidity, excess PMPs could not further reduce droplet size significantly.

#### 3.2.2. Physical Stability of HIPPEs

**Figure 6:**
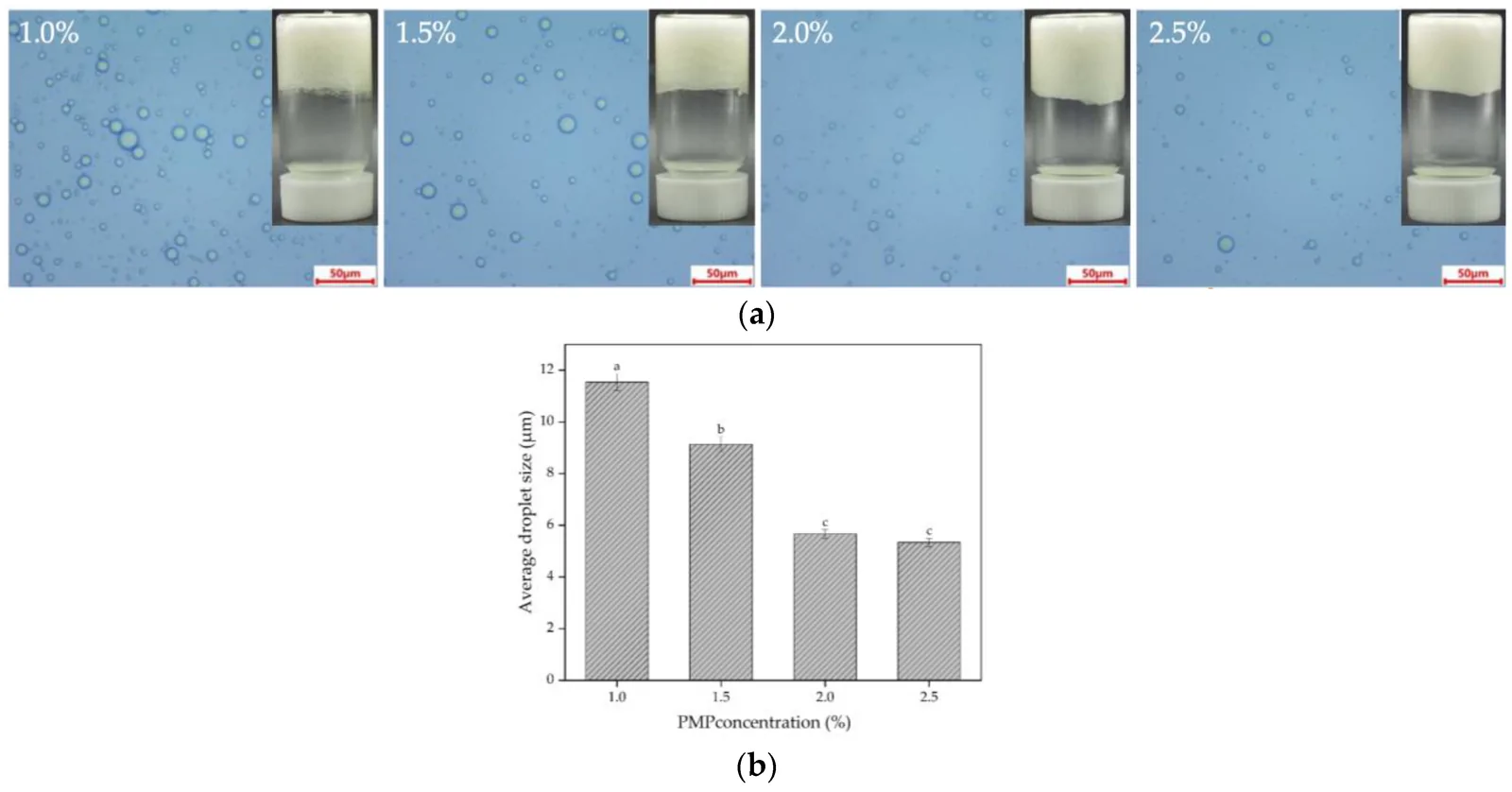
The macroscopic morphology and optical microscope images (**a**) and average droplet size (**b**) of HIPPEs after being stored at 4 °C for 30 days. One representative image from three independent batches is shown. Data are expressed as the mean ± SD of three independent HIPPE batches (*n* = 3). Different lowercase letters indicate significant differences among HIPPEs under different PMP concentrations (*p* < 0.05).

HIPPEs are commonly used in food products such as sausages and dairy products, which are typically stored at 4 °C. Therefore, HIPPEs stabilized with different concentrations of PMP were left undisturbed at 4 °C for 30 days to observe changes in emulsion appearance and average droplet size. As shown in Figure 6a, after 30 days of storage, the HIPPE with 1.0% PMP showed slight phase separation. In contrast, HIPPEs with PMP concentrations of 1.5%, 2.0%, and 2.5% showed no obvious oil-phase separation. They remained non-flowing when inverted, indicating good physical storage stability. Both HIPPEs with PMP concentrations of 1.0% and 1.5% exhibited a certain number of larger droplets. Compared to fresh HIPPEs, the average droplet sizes of all HIPPEs increased to varying degrees after 30 days of storage (Figure 6b). After 30 days, the average droplet sizes of HIPPEs stabilized at 1.0%, 1.5%, and 2.0% PMP were 11.54 μm, 9.13 μm, and 5.66 μm, respectively, and differed significantly (*p* < 0.05). The average droplet size of HIPPE with 2.5% PMP was 5.34 μm and did not differ significantly from that of HIPPE with 2.0% PMP. After 30 days of storage at 4 °C, HIPPEs with PMP concentrations of 1.5%, 2.0%, and 2.5% showed no visible oil-phase separation, and the average droplet size increased by less than 10% compared with the fresh samples, indicating good storage stability. In contrast, the HIPPE with 1.0% PMP showed slight phase separation and a larger increase in droplet size. Overall, the droplet sizes of the stored emulsions tended to increase, likely due to droplet aggregation or coalescence over time [30]. In HIPPEs with low PMP concentrations, the PMP content was insufficient, resulting in weak interfacial films that could not effectively stabilize the emulsion and leading to droplet aggregation and larger droplet formation. Increasing the PMP concentration provided sufficient particles to stabilize the emulsion, thereby reducing droplet aggregation and improving storage stability. At around 2.0% PMP concentration, enough PMP was present to cover the oil–water interface and form a stable interfacial film. Further increases in PMP concentration had little effect on average droplet size.

**Figure 7:**
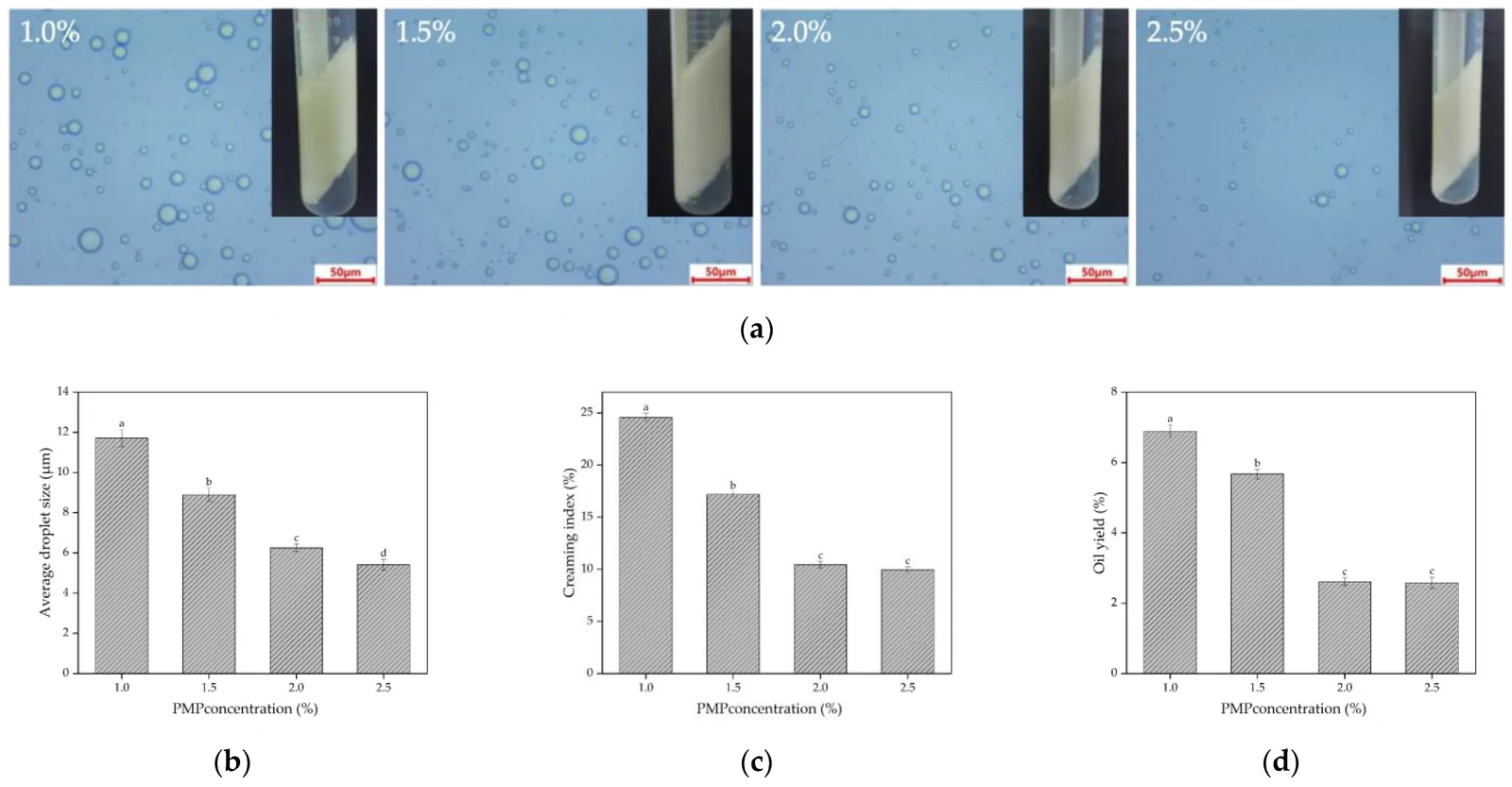
The macroscopic morphology and optical microscope images (**a**), average droplet size (**b**), creaming index (**c**), and oil yield (**d**) of HIPPEs after centrifugation. One representative image from three independent batches is shown. Data are expressed as the mean ± SD of three independent HIPPE batches (*n* = 3). Different lowercase letters indicate significant differences among HIPPEs under different PMP concentrations (*p* < 0.05).

To evaluate the stability of HIPPEs under centrifugal force, the centrifugal stability of the HIPPEs was analyzed by measuring droplet size, the creaming index, and oil yield. Figure 7a shows the macroscopic state and the microstructure of HIPPEs after centrifugation. Under the centrifugal force, the water phases in all HIPPEs were separated. After centrifugation, the oil phase separated in the HIPPE with 1.0% PMP and aggregated to form an oil layer. In HIPPE samples with concentrations of 1.5% to 2.5%, there was almost no oil-phase separation. Large droplets of HIPPEs appeared, possibly due to precipitation of the water phase under centrifugal force and the higher probability of droplet collision, which makes aggregation and coalescence easier. Figure 7b shows the average droplet size of HIPPEs after centrifugation. After centrifugation, the droplet size of all HIPPEs increased to a certain extent compared with the fresh ones. Still, the overall trend in particle-size change was consistent with that of freshly prepared HIPPEs. As shown in Figure 7c,d, as PMP concentration in the HIPPE system increased, both the creaming index and oil yield decreased. At PMP concentrations of 2.0% and 2.5%, the creaming index and oil yield showed no significant difference, indicating that the demulsification phenomenon was effectively suppressed. These results showed that higher PMP concentrations significantly enhanced the centrifugal stability of the HIPPE system and provided stronger resistance to centrifugal damage. This may be because, at low PMP content, only a thin interface layer formed at the oil–water interface. During centrifugation, this relatively weak interface layer was easily damaged, triggering demulsification and oil leakage. However, the internal structure of HIPPE droplets with a PMP concentration of 2.0% to 2.5% remained relatively intact after centrifugation. This may be because the increased PMP concentration caused many PMPs to pack closely at the oil–water interface, forming a denser, tougher interfacial layer. The interfacial layer may have buffered the impact of centrifugal force on the HIPPE system, significantly reducing the possibility of droplet aggregation and rupture, and thereby maintaining the relative stability of the emulsion structure.

**Figure 8:**
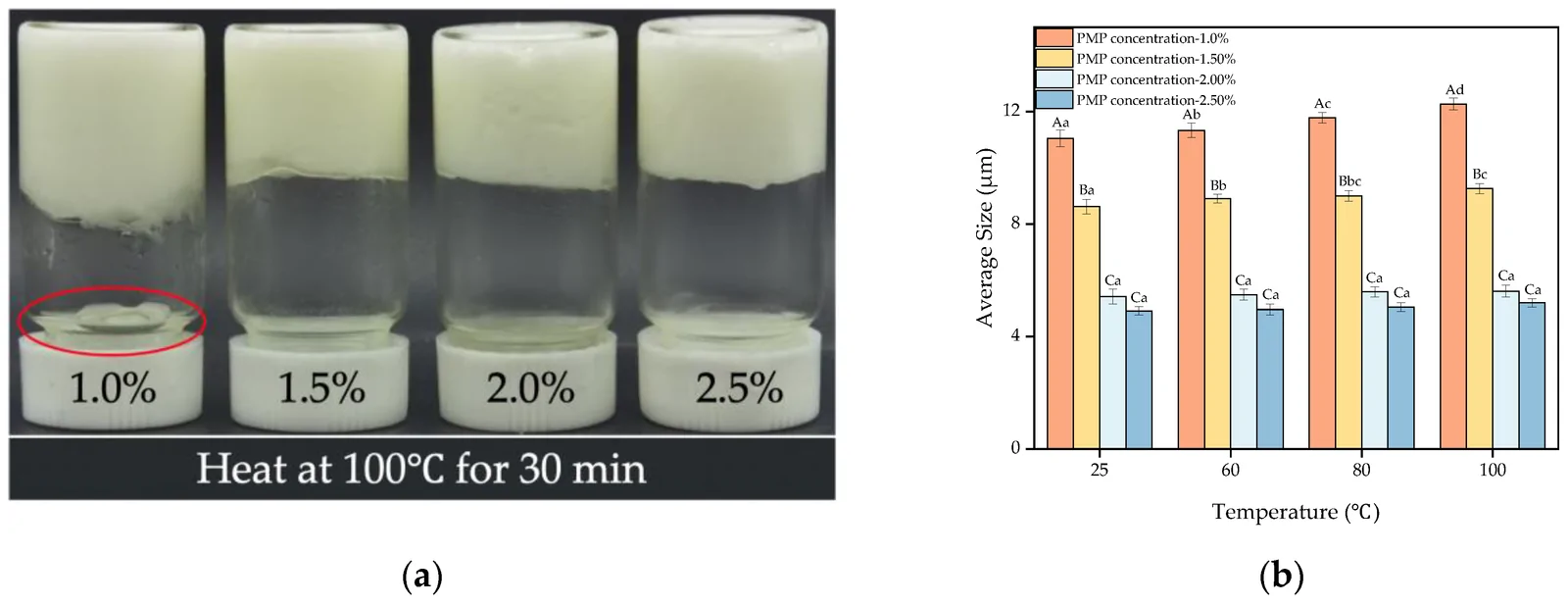
The macroscopic morphology (**a**) and average droplet size (**b**) of HIPPEs after heating at 25, 60, 80, and 100 °C for 30 min. One representative image from three independent batches is shown. Data are expressed as mean ± SD of three independent batches (*n* = 3). The batch effect was tested and found not to be statistically significant (*p* > 0.05). Different capital letters indicated significant differences among HIPPEs with different PMP concentrations within the same thermal treatment (*p* < 0.05). Different lowercase letters indicated significant differences among HIPPEs within the same PMP concentrations with different thermal treatments (*p* < 0.05, two-way ANOVA followed by Duncan’s multiple range test).

During food processing, HIPPEs can be used to produce sausages, meatballs, etc., which often require heating before consumption. To test the thermal stability of the HIPPEs, freshly prepared HIPPE samples were placed in water baths at 60 °C, 80 °C, and 100 °C for 30 min, and their macroscopic morphology and particle size were observed. Figure 8a shows the appearance of the sample after 30 min of heating at 100 °C. A small amount of oil-phase precipitation was observed in the 1.0% PMP concentration emulsion, while the other samples showed no significant changes. This indicated that when the PMP concentration was above 1.5%, HIPPEs remained stable during heat treatment. Figure 8b shows the droplet size of HIPPE after different temperature heat treatment. Two-way ANOVA revealed that both PMP concentration (F_3,32_ = 8.75 × 10^3^, *p* < 0.001) and temperature (F_3,32_ = 57.6, *p* < 0.001) significantly affected the average droplet size, and their interaction was also significant (F_9,32_ = 9.82, *p* < 0.001). Simple effects analysis showed that, at all tested temperatures, droplet size decreased significantly as PMP concentration increased from 1.0% to 2.0% (*p* < 0.05), while no significant difference was observed between 2.0% and 2.5% (*p* > 0.05) (Figure 8b). At a given PMP concentration, droplet size increased with increasing temperature. However, the extent of this increase depended on PMP concentration. At 1.0% and 1.5% PMP, average droplet size increased significantly from 25 °C to 100 °C (*p* < 0.05). In contrast, at 2.0% and 2.5% PMP, droplet size did not change significantly across the tested temperatures (*p* > 0.05), indicating that higher PMP concentrations improved the thermal stability of HIPPEs. These results suggest that sufficient PMP at the oil–water interface can form a dense interfacial layer that resists heat-induced droplet coalescence. At lower PMP concentrations, the interfacial layer may be incomplete, allowing droplet aggregation and coalescence upon heating. The number of PMPs that can adsorb and stabilize the oil–water interface continuously increased as the PMP concentration gradually increased. When the PMP concentration reached 2.0%, the PMP particle content in the system may have reached the critical value, and the formed interfacial adsorption layer was sufficient to maintain oil–water interface stability, thereby effectively inhibiting heat-induced changes in emulsion particle size. Similar findings have been reported for other protein-stabilized HIPPEs [14].

Based on the storage, centrifugal, and thermal stability results, HIPPE stabilized with 2.0% PMP was selected as an animal fat substitute for subsequent sausage experiments. At 2.0% PMP, sufficient PMPs adsorbed onto the oil–water interface to form a dense interfacial layer, resulting in a small droplet size (5.42 μm). Therefore, 2.0% PMP was considered sufficient to produce stable HIPPEs, since the difference between 2.0% and 2.5% PMP was not statistically significant for most indicators.

### 3.3. Characterization of the Sausages

#### 3.3.1. Appearance and Rheological Properties of Meat Batter

**Figure 9:**
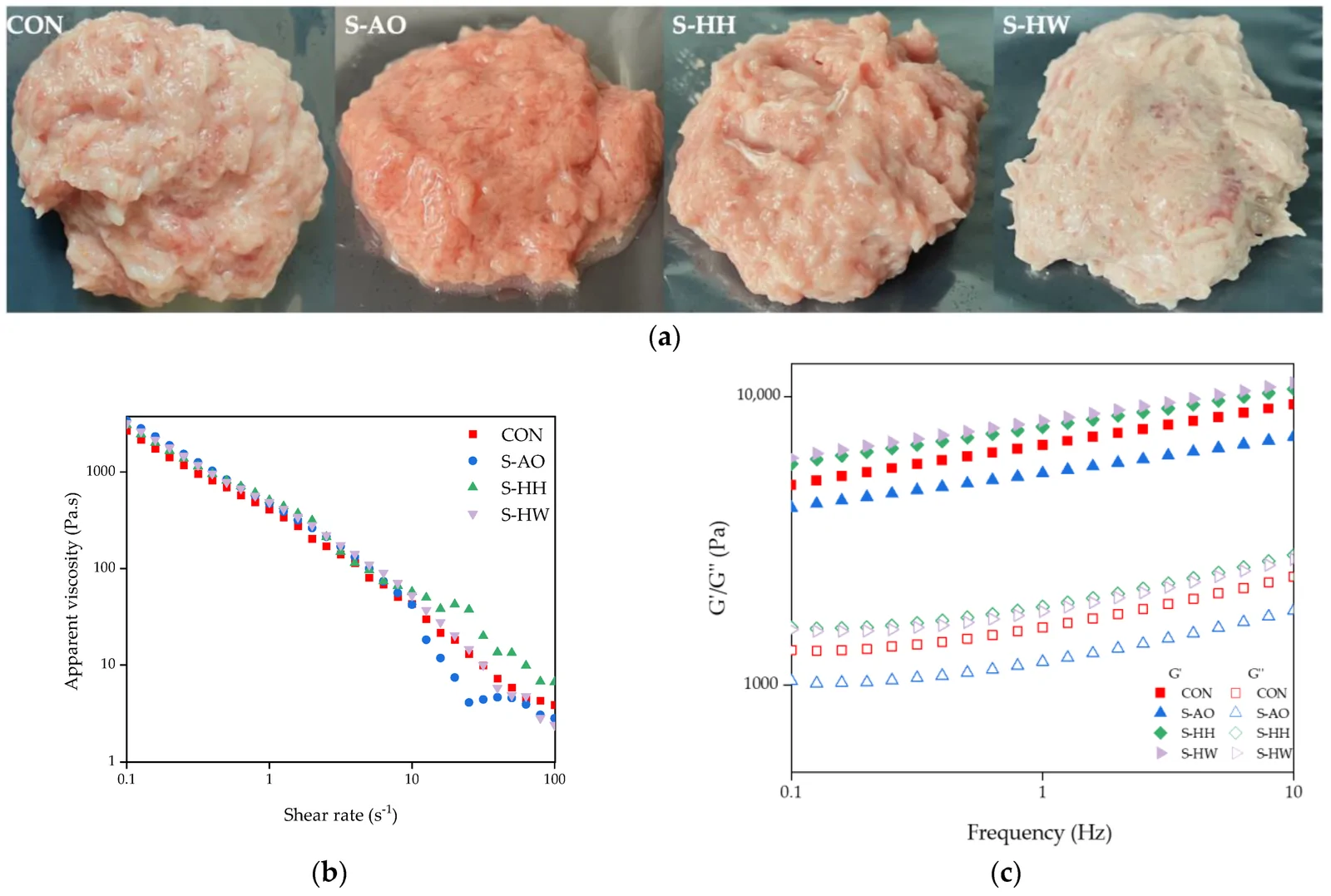
The appearance (**a**), viscosity profile (**b**), and frequency sweep (**c**) of meat batter with different formulas. One representative image from three independent batches is shown. CON: control sausage with sheep tail fat; S-AO: composition-matched, non-emulsified control for S-HW, in which almond oil and 2% PMP suspension were added directly; S-HH: sausage with HIPPE replacing half of the sheep tail fat (50% replacement); S-HW: sausage with HIPPE replacing the whole amount of sheep tail fat (100% replacement).

Figure 9 shows the appearance and rheological properties of the meat batter. The uniformity of meat batters with animal fat substituted with HIPPE improved (Figure 9a). Before filling the casing, the meat batter with almond oil showed a significant amount of liquid oozing out. When HIPPE was completely mixed with minced meat, the HIPPE oil droplets were smaller, making them easier to embed and distribute evenly within the protein matrix [21]. This resulted in a more uniform meat mixture. This indicates that HIPPE could improve the internal structure of meat batter and enhance product quality.

Figure 9b illustrates the apparent shear viscosity as a function of shear rate for raw meat batter. The apparent viscosity of all samples decreased with increasing shear rate, implying a pronounced shear-thinning behavior. This phenomenon may be attributed to the progressive disruption of weak protein crosslinks under elevated shear conditions, thereby reducing flow resistance and resulting in lower viscosity at higher shear rates. This result also indicated that the mixing rate should be controlled during meat batter processing. The apparent viscosity of raw batter with different formulas showed no significant differences, which may be due to hydrodynamic slip created by the high content of fat/oil in emulsion-type sausages.

G′ values of all raw meat batters were higher than G″ (Figure 9c), implying that the meat batter was gel-like. The G′ values of S-AO were slightly lower than those in the other groups. Increasing the fat substitution ratio increased the G′ values of the samples. This result was consistent with the appearance images, in which the minced meat in the S-AO sample showed significant collapse, and the molding property improved as the amount of HIPPE increased (from 50% to 100%). PMP adsorbed at the oil–water interface may interact with meat proteins, possibly contributing to the protein network structure. In addition, small HIPPE droplets dispersed in the protein-based minced meat may enhance the gel-like properties of the meat mixture. However, direct evidence for these interactions and dispersion of HIPPE droplets in sausages was not obtained in this study.

#### 3.3.2. Analysis of Proximate Composition and pH

The proximate compositions of the emulsion-type sausages are summarized in Table 2. The fat content decreased significantly from 21.59% in CON to 19.31% in S-AO and 19.62% in S-HH, and further to 18.90% in S-HW (*p* < 0.05). S-AO and S-HH did not differ significantly from each other, but both were significantly higher than S-HW (*p* < 0.05). The moisture content differed significantly among all four formulations (*p* < 0.05). Directly replacing sheep tail fat with almond oil (S-AO) significantly reduced the moisture content compared with CON (*p* < 0.05). In contrast, HIPPE substitution significantly increased the moisture content, with S-HW showing the highest value (*p* < 0.05). This finding was consistent with a previous study [4], which reported that soy protein isolate and sodium alginate-stabilized HIPPEs used as animal fat substitutes significantly decreased fat content and increased moisture content in emulsion-type sausages. Incorporating a gel-like HIPPE network could improve water-binding capacity [31]. Thus, the gel-like rheological properties of HIPPEs maintained the moisture trapped within the protein matrix and emulsion droplets, preventing phase separation before/after thermal treatment. HIPPE substitution reduced total fat content and increased moisture content in the sausages (Table 2). The oil phase in HIPPE was almond oil, which has been reported to contain a high proportion of monounsaturated and polyunsaturated fatty acids, with oleic acid as the main component [32]. Previous studies reported its protective effects in cardiovascular risk management, neuroprotection, oxidative stress reduction, glucose homeostasis, and many dermatologic applications [32,33]. However, the fatty acid composition, energy value, or health-related outcomes of the sausages were not determined in this study, and the potential nutritional implications of this substitution require further investigation. The amount of PMPs in HIPPE was negligible and insufficient to affect the protein and mineral content dominated by the meat matrix (Table 2). These findings indicated that PMP-stabilized HIPPE could be used as a viable fat substitute in fat-reformulated emulsion-type sausages.

pH is a key indicator for emulsion-type sausages, as it reflects myofibrillar protein solubilization and batter stability. In Table 2, the pH ranged from 6.28 to 6.35. CON had the highest pH (6.35), while S-AO and S-HW had the lowest pH (6.28). S-HH showed an intermediate pH (6.33) that did not differ significantly from CON. Although the pH differences were statistically significant in some comparisons, all values remained within an acceptable range for emulsion-type sausage products. This result aligns with previous findings that introducing HIPPEs induced a similar pH decline [4].

#### 3.3.3. Cooking Loss, WHC, and Emulsion Stability

Cooking loss is a critical parameter for evaluating emulsion-type sausages, as it directly reflects their water-holding ability and oil-binding capacity during cooking [4]. As shown in Table 3, when the proportion of HIPPE substitution increased, it was beneficial for reducing the cooking loss of emulsion-type sausages. At 50% fat substitution, the cooking loss of S-HH decreased compared with the control group, but the difference was not statistically significant. When animal fat was completely replaced with HIPPE, the cooking loss of S-HW significantly decreased from 5.03% to 3.20%, reducing the cooking loss by 36%. However, the cooking loss of sausages with animal fat replaced with liquid vegetable oil (almond oil) (which were the same as the S-HW formula) was the highest (7.46%), which was 2.43% higher than that of the control group sausages. A previous study reported that salmon protein-stabilized HIPPEs could reduce the cooking loss of emulsion-type sausages by replacing animal fat [19]. That result was consistent with this study. Three possible explanations may account for this result. First, HIPPE had high centrifugal and thermal stability (Figure 7 and Figure 8), which may help maintain sausage structure. Second, during heating, myofibrillar proteins in the meat batter may interact with PMP on the oil–water interface of HIPPE, and thermal denaturation may expose hydrophobic groups. These proteins may contribute to a more compact three-dimensional gel network structure through hydrophobic interactions and physical entanglement. Third, when HIPPEs are added to emulsion-type sausages, they may undergo double emulsification, resulting in uniform distribution within the protein matrix of the meat batter and further reducing cooking loss.

WHC indicates the integrity of the three-dimensional gel network within the sausage. Directly adding almond oil significantly reduced the WHC of sausages (*p* < 0.05), while HIPPE significantly increased the WHC of sausages (Table 3). Higher WHC led to decreased cooking loss. These results were consistent with previous findings [34]. The S-AO group served as a composition-matched, non-emulsified control for S-HW. Both groups contained the same total PMP (0.4 g) and similar total water content (~40 g), with only a small difference in almond oil content (60 g in S-AO vs. approximately 58.6 g in S-HW, a difference of approximately 2.3%). Despite this small difference, S-HW showed significantly higher WHC and lower cooking loss than S-AO, suggesting that the HIPPE structure itself, rather than the small difference in oil content, was primarily responsible for the improved quality. This observation may be due to HIPPE enhancing the gel properties of the sausages. At the same time, liquid almond oil could not retain internal moisture effectively, resulting in aqueous separation under centrifugal force.

During heat processing, meat products are susceptible to water loss, fat release, and structural loosening, which adversely affect overall quality. Emulsion stability, determined by measuring TEF and fat in TEF, is often used to characterize the ability of sausages to retain water and fat. In Table 3, TEF and fat in TEF of the control sample were 19.31% and 14.27%, respectively. Directly adding almond oil significantly increased TEF and fat in TEF because it loosened the sample structure. Both TEF and fat in TEF of emulsion-type sausages with HIPPE were lower than those of the control group. These reductions exhibited a positive dependence on the HIPPE substitution level, implying that replacing animal fat with HIPPEs effectively improved the emulsion stability of emulsion-type sausages. Wang et al. also found that increasing the substitution ratio of the soy protein isolate/sodium alginate-stabilized HIPPE as pork backfat significantly reduced TEF and FAT of sausages [4], similar to the results of this study. Furthermore, possible interactions between PMP adsorbed at the oil–water interface of HIPPE and meat proteins may contribute to a denser structure in emulsion-type sausages [35], thereby enhancing emulsion stability. However, direct evidence for these interactions was not obtained in this study.

#### 3.3.4. Color and Textural Profile Analysis

Color parameters, which reflect the internal structure of sources and forms of oil/fat, are key indicators for evaluating emulsion-type sausages. The color values of the emulsion-type sausages are shown in Table 4. There were no significant differences in a* values (redness) among the sausages (*p* > 0.05). Compared with the control group, the L* (lightness) and b* (yellowness) values of the emulsion-type sausages with whole animal fat replaced by almond oil were significantly decreased (*p* < 0.05). As HIPPE substituted the animal fat, the L* increased and b* decreased compared with the control group. However, the differences were statistically significant in S-HW (*p* < 0.05) but not in S-HH (*p* > 0.05). The increased lightness of sausages with HIPPE may be related to smaller oil droplets in HIPPE, which could increase light scattering at the surface. In addition, the WHC of emulsion-type sausages with HIPPE was higher than that of the control sausages (Table 3), which could increase light scattering at the surface. This result was consistent with a previous study [21]. However, this study did not directly establish the relationship between WHC, water distribution, droplet size, and CIELAB lightness, and further investigation is needed. The changes in redness and yellowness are different from the results reported in the previous study [21]. These differences likely reflected variations in formula ingredients, especially the oil components, across studies. In general, replacing animal fat with HIPPE can improve the visual characteristics of emulsified sausages and may affect panelist acceptability.

The ΔE* value indicates the total color difference between each sample and the control. The ∆E* values for sausages increased significantly as the HIPPE substitution ratio increased. The ∆E* values for sausages with almond oil or HIPPE replacing 100% of animal fat exceeded 3 CIELAB units, resulting in distinguishable color changes to the human eye. The ∆E* value of S-HH was 1.0, indicating subtle differences that can only be detected by trained professionals. This result indicated that replacing 50% of animal fat with HIPPE had no significant effect, but replacing whole animal fat with almond oil and HIPPE did affect sausage color. However, along with the change in L* value, we speculated that the effect of HIPPE may be positive.

Texture, relating to bite, chewiness, and mouthfeel, is a key determinant of consumer acceptability. Table 4 shows the texture properties of emulsion-type sausages. Three independently manufactured batches were used for TPA (*n* = 3). For each batch, three cylindrical samples were measured, and their mean was used as a single data point. Compared with the control sample, the S-AO group showed an obvious reduction in texture. However, the HIPPE increased the hardness, springiness, and chewiness of the emulsion-type sausages. This is related to the higher G′ of S-HH and S-HW (Figure 9). The cohesiveness of emulsion-type sausages was not affected by the HIPPE replacement. Three factors mainly drive the increase in texture parameters. First, HIPPE had smaller oil droplets, which can be effectively distributed as a filler within the sausage gel network. Second, heat treatment may promote possible interactions, including hydrophobic interactions, electrostatic interactions, thermal gelation, and physical entanglement, between PMP adsorbed at the oil–water interface of HIPPE and proteins in the meat batter, which may contribute to a more stable three-dimensional network structure. Third, HIPPE had high viscoelasticity, which increased the mechanical resistance of the gel network. This result aligned with findings reported by Yan et al. [21]. Therefore, the sausages with HIPPE substitution exhibited excellent textural properties.

The possible interactions between PMP and meat proteins may involve several mechanisms. Hydrophobic interactions may occur when thermal denaturation exposes hydrophobic groups normally buried within the protein core. Electrostatic interactions may be affected by pH and ionic strength, although the pH of the sausages remained within a narrow range (6.28–6.35). Thermal gelation may contribute to network formation through the unfolding and subsequent aggregation of myofibrillar proteins. Physical entanglement may occur between PMP at the interface and the continuous protein matrix. However, these mechanisms should be considered as possible explanations rather than established mechanisms.

#### 3.3.5. Sensory Evaluation

**Figure 10:**
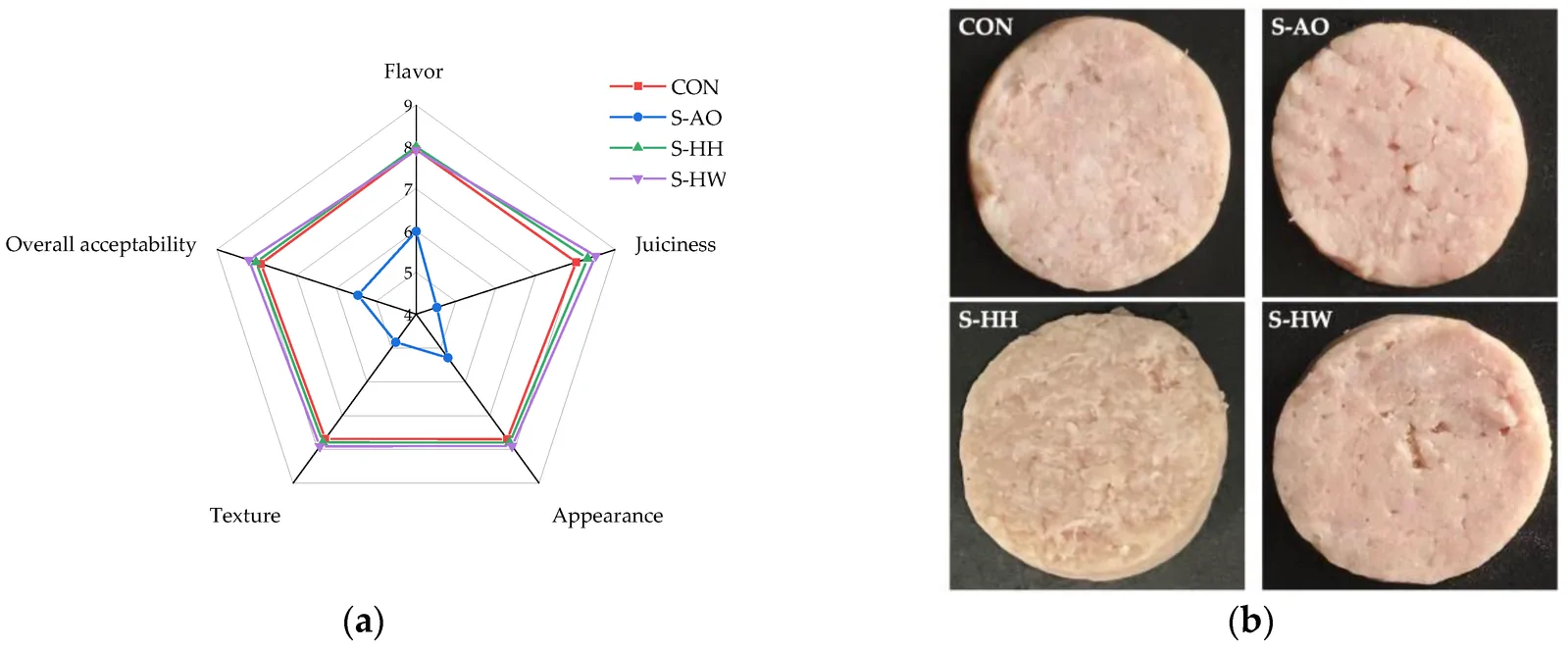
(**a**) Sensory evaluation and (**b**) apparent images of sausages. CON: control sausage with sheep tail fat; S-AO: composition-matched, non-emulsified control for S-HW, in which almond oil and 2% (*w*/*v*) PMP suspension were added directly; S-HH: sausage with HIPPE replacing half of the sheep tail fat (50% replacement); S-HW: sausage with HIPPE replacing the whole amount of sheep tail fat (100% replacement). Sensory data are expressed as mean ± SD of 40 panelists (*n* = 40). Detailed mean ± SD values and significance letters are provided in Table S1. One representative image from three independent batches is shown.

Since sensory acceptability is important for the commercial success of fat-reformulated meat products, sensory analysis is an essential tool for evaluating the acceptability of emulsion-type sausages. The sensory scores are shown in Figure 10a, and the corresponding mean ± SD values and significance letters are provided in Table S1. The sensory evaluation scores of S-AO ranged from 4 to 6, significantly lower than those of 7 to 9, indicating the need to pre-emulsify the vegetable oil before using it to replace animal fat in sausages. All sensory attribute scores for CON, S-HH, and S-HW exceeded 7 points, indicating that substituting sheep tail fat with HIPPE preserved emulsion-type sausage quality at levels that panelists would find acceptable. These attributes were related to the greasy appearance and loose structure in the apparent images (Figure 10b). Flavor scores showed no significant differences (*p* > 0.05) when comparing HIPPE as a fat substitute with the control group (CON). This result may reflect the different cultural backgrounds and previous dietary experiences of evaluators, which are known to influence individual preferences for characteristic mutton flavor. Compared with CON, the juiciness score of S-HH and S-HW significantly increased. This sensory attribute was consistent with increased moisture content and decreased cooking loss, TEF, and fat in TEF, and may have enhanced instrumental texture parameters. In addition, the appearance, texture, and overall acceptability of S-HH and S-HW were significantly higher than those of the sausage with sheep tail fat (*p* < 0.05). Increasing HIPPE substitution levels significantly enhanced the sensory score, meaning that HIPPE could substitute animal fat in emulsion-type sausages and maintain better quality. These findings confirmed that the PMP-stabilized HIPPE could serve as a promising fat substitute, thereby achieving enhanced sensory acceptability within the study panel.

## 4. Conclusions

This study demonstrated that PMP-stabilized emulsions can serve as effective fat substitutes in emulsion-type sausages. AFM analysis confirmed that PMP can form nanoscale particles, and SDS-PAGE and CLSM analyses confirmed the adsorption of PMP at the oil–water interface. These results are consistent with Pickering-type stabilization. Increasing PMP concentration from 0.5% to 2.0% progressively enhanced the physical storage (over a 30-day period), centrifugal, and thermal stability of HIPPEs. HIPPE with 2.0% PMP was considered sufficient to produce stable HIPPEs, since the difference between 2.0% and 2.5% PMP was not statistically significant for most indicators. HIPPE substitution improved the rheological properties, WHC, and emulsion stability of the sausages, which may be related to a more compact gel network, although the protein network was not directly characterized microscopically in this study. Complete replacement of animal fat with HIPPEs reduced cooking loss from 5.03% to 3.20% (a 36% reduction), while hardness and chewiness increased notably. The S-AO group showed significantly lower WHC and higher cooking loss than S-HW, indicating that the HIPPE structure, rather than the small difference in ingredient composition, was primarily responsible for the improved quality. Sensory evaluation by a panel of food-science students indicated that all HIPPE-substituted sausages were well accepted within the study panel, with overall acceptability scores above 7 out of 9. These quality improvements may be partially attributed to the PMP-rich interfacial layer at the oil–water interface and possible heat-induced interactions between PMP and meat proteins. These interactions may contribute to a more compact gel network, although the protein network was not directly characterized microscopically in this study. The interpretation of a denser network is therefore presented as a hypothesis consistent with the rheological, WHC, emulsion-stability, and texture results, rather than as a directly demonstrated structural change. Almond oil may contribute to the nutritional profile of the final product, although this was not directly evaluated in the present study. Thus, substituting animal fat with PMP-stabilized HIPPEs can produce fat-reformulated emulsion-type sausages with reduced total fat content and improved technological quality. Almond oil is rich in unsaturated fatty acids and has been reported to exhibit potential health benefits. However, the oxidation stability, nutritional composition, or health implications of the final sausages were not evaluated in this study. Future work should determine these aspects of the final products.

## Figures and Tables

**Table 1:** Formula of emulsion-type sausages using almond oil/HIPPE as a substitute for animal fat (sheep tail fat).

Ingredients (g)	CON	S-AO	S-HH	S-HW
Sheep lean meat	180.00	180.00	180.00	180.00
Sheep tail fat	60.00	-	30.00	-
Almond oil	-	60.00	-	-
2% *w*/*v* PMP suspension	-	20.00	-	-
Ice water	40.00	20.00	30.00	20.00
HIPPE	-	-	40.00	80.00
Starch	10.00	10.00	10.00	10.00
Salt	5.00	5.00	5.00	5.00
White pepper powder	1.00	1.00	1.00	1.00
Ginger powder	0.40	0.40	0.40	0.40
Sodium tripolyphosphate	1.00	1.00	1.00	1.00
Ascorbic acid sodium	0.16	0.16	0.16	0.16

CON: control sausage with sheep tail fat; S-AO: composition-matched, non-emulsified control for S-HW, in which almond oil and 2% (*w*/*v*) PMP suspension were added directly; S-HH: sausage with HIPPE replacing half of the sheep tail fat (50% replacement); S-HW: sausage with HIPPE replacing the whole amount of sheep tail fat (100% replacement). The replacement levels of 50% and 100% refer to the replacement of sheep tail fat by an equivalent mass of almond oil on a nominal basis. HIPPE was prepared at an oil-to-aqueous phase volume ratio of 3:1 (*v*/*v*), corresponding to an oil mass fraction of approximately 73.2% (*w*/*w*). Therefore, 40 g of HIPPE contained approximately 29.3 g of almond oil, 9.8 g of water, and 0.2 g of PMP; 80 g of HIPPE contained approximately 58.6 g of almond oil, 19.6 g of water, and 0.4 g of PMP. Ice water was adjusted so that the total formulation mass was constant across all treatments. The S-AO and S-HW groups had the same total formulation mass (297.56 g), similar total PMP content (~0.4 g), and similar total water content (~40 g). The almond oil content was 60 g in S-AO and approximately 58.6 g in S-HW; the small difference (approximately 2.3%) arose from the density difference between almond oil and the aqueous phase.

**Table 2:** The proximate composition and pH of the cooked sausages (mean ± SD; *n* = 3).

Treatments	CON	S-AO	S-HH	S-HW
Fat (%)	21.59 ± 0.11 ^a^	19.31 ± 0.23 ^b^	19.62 ± 0.21 ^b^	18.90 ± 0.10 ^c^
Protein (%)	13.25 ± 0.14 ^a^	13.39 ± 0.17 ^a^	13.58 ± 0.10 ^a^	13.48 ± 0.14 ^a^
Ash (%)	3.81 ± 0.04 ^a^	3.84 ± 0.07 ^a^	3.85 ± 0.07 ^a^	3.80 ± 0.04 ^a^
Moisture (%)	58.09 ± 0.10 ^c^	56.32 ± 0.15 ^d^	62.05 ± 0.13 ^b^	63.88 ± 0.18 ^a^
pH	6.35 ± 0.01 ^a^	6.28 ± 0.04 ^bc^	6.33 ± 0.02 ^ab^	6.28 ± 0.02 ^c^

CON: control sausage with sheep tail fat; S-AO: composition-matched, non-emulsified control for S-HW, in which almond oil and 2% (*w*/*v*) PMP suspension were added directly; S-HH: sausage with HIPPE replacing half of the sheep tail fat (50% replacement); S-HW: sausage with HIPPE replacing the whole amount of sheep tail fat (100% replacement). Data are expressed as mean ± SD of three independent batches (*n* = 3). The batch effect was tested and found not to be statistically significant (*p* > 0.05). Different lowercase letters in the same row indicate significant differences among sausages with different formulations (*p* < 0.05, one-way ANOVA followed by Duncan’s multiple range test).

**Table 3:** The cooking loss, WHC, and emulsion stability of sausages with different formulas (mean ± SD; *n* = 3).

Treatments	CON	S-AO	S-HH	S-HW
Cooking loss (%)	5.03 ± 0.28 ^b^	7.46 ± 0.20 ^c^	4.99 ± 0.01 ^b^	3.20 ± 0.11 ^a^
WHC (%)	85.98 ± 0.94 ^c^	75.05 ± 4.17 ^d^	89.33 ± 0.14 ^b^	92.46 ± 0.63 ^a^
TEF (%)	19.31 ± 0.71 ^b^	24.36 ± 3.01 ^a^	18.68 ± 0.41 ^b^	7.37 ± 1.96 ^c^
Fat in TEF (%)	14.27 ± 1.71 ^b^	43.57 ± 9.54 ^a^	10.05 ± 1.69 ^c^	5.60 ± 1.23 ^d^

CON: control sausage with sheep tail fat; S-AO: composition-matched, non-emulsified control for S-HW, in which almond oil and 2% (*w*/*v*) PMP suspension were added directly; S-HH: sausage with HIPPE replacing half of the sheep tail fat (50% replacement); S-HW: sausage with HIPPE replacing the whole amount of sheep tail fat (100% replacement). Data are expressed as mean ± SD of three independent batches (*n* = 3). The batch effect was tested and found not to be statistically significant (*p* > 0.05). Different lowercase letters in the same row indicate significant differences among sausages with different formulations (*p* < 0.05, one-way ANOVA followed by Duncan’s multiple range test).

**Table 4:** Internal color parameters and texture profile attributes of the cooked sausages with different formulas (mean ± SD; *n* = 3).

Treatments	CON	S-AO	S-HH	S-HW
L*	60.16 ± 0.24 ^b^	58.44 ± 0.25 ^c^	60.28 ± 0.49 ^b^	64.63 ± 0.82 ^a^
a*	10.24 ± 0.62 ^a^	9.03 ± 0.54 ^a^	10.21 ± 0.62 ^a^	9.75 ± 0.76 ^a^
b*	12.54 ± 0.70 ^a^	10.41 ± 0.82 ^b^	12.09 ± 0.91 ^ab^	12.04 ± 0.84 ^a^
ΔE*	--	3.01 ± 0.92 ^a^	1.04 ± 0.39 ^b^	4.63 ± 0.70 ^a^
Hardness (N)	45.16 ± 2.55 ^c^	37.26 ± 4.55 ^d^	54.47 ± 2.65 ^b^	65.00 ± 3.80 ^a^
Cohesiveness (ratio)	0.58 ± 0.13 ^a^	0.57 ± 0.03 ^a^	0.43 ± 0.07 ^a^	0.57 ± 0.15 ^a^
Springiness (mm)	3.26 ± 0.42 ^bc^	2.41 ± 0.04 ^dc^	5.42 ± 0.57 ^a^	4.17 ± 0.77 ^b^
Chewiness (N·mm)	82.98 ± 6.49 ^b^	50.96 ± 3.14 ^c^	127.24 ± 23.83 ^a^	148.87 ± 12.36 ^a^

CON: control sausage with sheep tail fat; S-AO: composition-matched, non-emulsified control for S-HW, in which almond oil and 2% (*w*/*v*) PMP suspension were added directly; S-HH: sausage with HIPPE replacing half of the sheep tail fat (50% replacement); S-HW: sausage with HIPPE replacing the whole amount of sheep tail fat (100% replacement). Data are expressed as mean ± SD of three independent batches (*n* = 3). Within each batch, measurements were performed in triplicate, and the mean was used as a single data point. The batch effect was tested and found not to be statistically significant (*p* > 0.05). Different lowercase letters in the same row indicate significant differences among sausages with different formulations (*p* < 0.05, one-way ANOVA followed by Duncan’s multiple range test).

## Data Availability

The original contributions presented in this study are included in the article/Supplementary Material. Further inquiries can be directed to the corresponding author.

## Supplementary Materials

The following supporting information can be downloaded at: https://www.mdpi.com/article/10.3390/foods15213820/s1, Table S1: Sensory evaluation scores (mean ± SD) of emulsion-type sausages with different formulations.

## Abbreviations

The following abbreviations are used in this manuscript:

HIPPE	High Internal Phase Pickering Emulsion
PMP	Pigeon Myofibrillar Protein
CON	Control Sausage with Sheep Tail Fat
S-AO	Sausage with Almond Oil Directly Replacing Sheep Tail Fat
S-HH	Sausage with HIPPE Replacing Half of the Sheep Tail Fat (50% Replacement)
S-HW	Sausage with HIPPE Replacing the Whole Amount of the Sheep Tail Fat (100% Replacement)
G′	Storage Modulus
G″	Loss Modulus
WHC	Water-Holding Capacity
TEF	Total Expressible Fluid
