# Supplementary material for: Application of Pigeon Myofibrillar Protein-Stabilized High Internal Phase Pickering Emulsions as a Sheep Tail Fat Substitute in Emulsion-Type Sausages
Source: Foods. 2026 Oct 26;15(21):3820. doi: 10.3390/foods15213820 (PMC13762690; doi:10.3390/foods15213820)
Supplement: Supplementary file 1 [file foods-15-03820-s001.zip › foods-4550948-supplementary.pdf]

Table S1. Sensory evaluation scores (mean ± SD) of emulsion-type sausages with different formulations.

Parameter evaluated	CON	S-AO	S-HH	S-HW
Flavor	7.94 ± 0.79 ^{a}	5.98 ± 0.61 ^{b}	8.00 ± 0.66 ^{a}	7.92 ± 0.58 ^{a}
Juiciness	8.02 ± 0.68 ^{b}	4.52 ± 0.84 ^{c}	8.31 ± 0.71 ^{a}	8.51 ± 0.65 ^{a}
Appearance	7.70 ± 0.63 ^{b}	5.29 ± 0.92 ^{c}	7.80 ± 0.68 ^{ab}	7.90 ± 0.60 ^{a}
Texture	7.69 ± 0.66 ^{b}	4.83 ± 0.95 ^{c}	7.79 ± 0.72 ^{ab}	7.91 ± 0.74 ^{a}
Overall acceptability	7.90 ± 0.58 ^{b}	5.46 ± 0.82 ^{c}	8.02 ± 0.64 ^{ab}	8.20 ± 0.61 ^{a}

CON: control sausage with sheep tail fat; S-AO: composition-matched, non-emulsified control for S-HW, in which almond oil and 2% (w/v) PMP suspension were added directly; S-HH: sausage with HIPPE half of the sheep tail fat (50% replacement); S-HW: sausage with HIPPE replacing the whole amount of sheep tail fat (100% replacement). Data are expressed as mean ± SD of 40 panelists (n = 40). Different lowercase letters in the same row indicate significant differences among formulations ($P < 0.05$, mixed-effects model with Bonferroni correction).
